# Controversies in modern evolutionary biology: the imperative for error detection and quality control

**DOI:** 10.1186/1471-2164-13-5

**Published:** 2012-01-04

**Authors:** Francisco Prosdocimi, Benjamin Linard, Pierre Pontarotti, Olivier Poch, Julie D Thompson

**Affiliations:** 1 Department of Integrated Structural Biology, IGBMC (Institut de Génétique et de Biologie Moléculaire et Cellulaire) CNRS/INSERM/Université de Strasbourg, 1 rue Laurent Fries, Illkirch, F-67404, France; 2 Medical Biochemistry Department, Federal University of Rio de Janeiro, Avenida Carlos Chagas Filho 373, Rio de Janeiro, 21941-902, Brazil; 3 UMR-CNRS 6632 Evolution Biologique et Modélisation, Université de Provence, 3, Place Victor Hugo, Marseille, 13331, France

**Keywords:** gene duplication, asymmetric evolution, gene prediction, error detection, quality control

## Abstract

**Background:**

The data from high throughput genomics technologies provide unique opportunities for studies of complex biological systems, but also pose many new challenges. The shift to the genome scale in evolutionary biology, for example, has led to many interesting, but often controversial studies. It has been suggested that part of the conflict may be due to errors in the initial sequences. Most gene sequences are predicted by bioinformatics programs and a number of quality issues have been raised, concerning DNA sequencing errors or badly predicted coding regions, particularly in eukaryotes.

**Results:**

We investigated the impact of these errors on evolutionary studies and specifically on the identification of important genetic events. We focused on the detection of asymmetric evolution after duplication, which has been the subject of controversy recently. Using the human genome as a reference, we established a reliable set of 688 duplicated genes in 13 complete vertebrate genomes, where significantly different evolutionary rates are observed. We estimated the rates at which protein sequence errors occur and are accumulated in the higher-level analyses. We showed that the majority of the detected events (57%) are in fact artifacts due to the putative erroneous sequences and that these artifacts are sufficient to mask the true functional significance of the events.

**Conclusions:**

Initial errors are accumulated throughout the evolutionary analysis, generating artificially high rates of event predictions and leading to substantial uncertainty in the conclusions. This study emphasizes the urgent need for error detection and quality control strategies in order to efficiently extract knowledge from the new genome data.

## Background

High throughput genomics technologies are now providing the raw data for genome-level or systems-level studies [1]. At the same time, the avalanche of data also poses many new challenges. The shift to genome scale studies in evolutionary biology, for instance, has led to many interesting, but often controversial studies. Many branches in the Tree of Life are still the subject of intense discussions, and simply adding more sequences has not resolved the inconsistencies [2]. In prokaryotes, phylogenetic incongruencies are often assumed to be the result of lateral gene transfers, but the frequency of these events has been challenged recently [3,4]. In eukaryotes, the ancestral relationships between the major eukaryotic kingdoms [5-8], as well as many more recent clades such as fish or mammalian [9-11], are also hotly debated. It has been suggested that at least some of the conflicting results from evolutionary analyses are due to differences in the models and methodologies used to test the original hypotheses, e.g. [12,13], as well as errors in the input sequences [2].

High throughput biological datasets are notoriously incomplete [14-16], noisy and inconsistent and DNA or protein sequences are no exception. The DNA sequences produced by next generation sequencing (NGS) technologies or low-coverage assemblies pose particular problems [17,18]. A number of recent studies have investigated the rate of errors in these new genome sequences and their impact on the accuracy of downstream analyses [19-22]. In the context of proteome studies, the DNA sequencing errors are further confounded by inaccuracies in the delineation of the protein-coding genes. Coding regions are mostly predicted by automatic methods, but the relationship between genes, transcripts and proteins is complex and automated genome annotation is not completely accurate. Thus, ten years after the publication of the human genome, the exact number of human protein-coding genes is still unknown [23]. Furthermore, recent analyses have shown that, even for those genes that have been identified, the complete exon/intron structure is correctly predicted for only about 50-60% of them [24-26]. In eukaryotic genomes, the situation is also complicated by widespread alternative splicing events, which affects more than 92-94% of multi-exon human genes [27].

To what extent do these quality issues affect our understanding of the evolutionary events shaping modern organisms? Although sequence errors are essentially ignored in most genome-scale analyses, some studies have addressed certain aspects of this question. For example, Hubisz and coworkers [19] investigated the impact of DNA sequencing errors in low-coverage genome assemblies on inferred rates and patterns of insertion/deletion and substitution on the mammalian phylogeny. Schneider et al. [28] showed that the estimated amount of positively selected genes in genome scale analyses may be inflated by the presence of unreliable sequences.

Here, we have investigated the impact of erroneous protein sequences, resulting from either DNA sequencing errors or inaccurate prediction of exon/intron structures, on evolutionary analyses and the detection of important genetic events. We concentrated specifically on duplication events, which are known to be an important source of functional diversity [29-32] and where there has been a great deal of debate about the long term fate of duplicated genes. Two main models have been proposed for the evolution of novel gene function associated with gene duplication. The neofunctionalization model predicts the evolution of a new function in one of the duplicates, with accelerated evolution of the deconstrained copy compared with the copy that retains the ancestral function. The subfunctionalization model implies the division of the ancestral functions among the duplicates and does not make any prediction about the symmetry or asymmetry of sequence evolution. Although individual cases of both modes of evolution have been reported, the relative frequency of the different scenarios in nature is still not clear [12,33,34].

To some extent, the evolutionary fate of duplicated genes depends on the duplication mechanism. After tandem duplications or large-scale (e.g. whole-chromosome or whole-genome) duplications, both gene copies retain the same genome context. In contrast, after segmental duplications or retrotranspositions, one of the gene copies retains the ancestral genome position while the other copy is relocated elsewhere. It is generally expected that the gene copy that retains the genome context will be more conserved, and thus will be more likely to retain the ancestral functions [35]. The hypothesis is that newly duplicated genes that have been transposed to new chromosomal locations experience a new genomic and epigenetic environment, modifying the expression and/or function of the genes.

**Figure 1:**
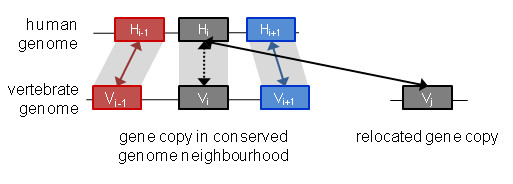
**Evolutionary scenario involving asymmetrical evolution after duplication (AED)**. A schematic view of the AED events included in this study. Using the human gene H_i _as a reference, homologs are detected in each vertebrate genome that maintain the same genome neighborhood as the human gene. At the same time, the homologs from each genome with the highest similarity to the human reference gene are identified (full arrows indicate similarity homologs and dashed arrows indicate syntenic homologs). We then selected AED events where the relocated similarity homolog has evolved significantly faster than the local syntenic homolog.

In this work, we have searched for duplication events that contradict this hypothesis, in order to quantify the effect of protein sequence errors on our ability to accurately identify unusual evolutionary histories. The goal was not to identify an exhaustive list of duplications, but to establish a reliable test set of events that could be used for the error analysis. Using the well-studied human genome as a reference, we identified 114,680 homologs in 13 high coverage vertebrate genomes from the Ensembl [36] database that were located in a region with local synteny (Figure 1). We then identified 688 cases where another homolog of the reference human gene was found elsewhere in the vertebrate genome with significantly higher sequence similarity than the syntenic homolog. In other words, we identified 688 gene triplets, composed of one human reference gene and two corresponding gene copies from another vertebrate genome (the local "syntenic homolog" and the remote "highest similarity homolog"), that might indicate putative asymmetric evolution after duplication (AED) events where the less similar gene copy retained the ancestral gene-neighbourhood. To determine what proportion of these putative AED events may be due to erroneous protein sequences (resulting from either DNA sequencing errors or badly predicted protein coding regions), we identified potential sequence errors in the gene triplets and showed that the majority (57%) of detected AED events are in fact false positives. A Gene Ontology (GO) functional analysis highlighted a number of GO categories that are over-represented in the true positive gene set, which were masked before filtering of the erroneous sequences.

## Results

### Estimation of sequence error rates

**Figure 2:**
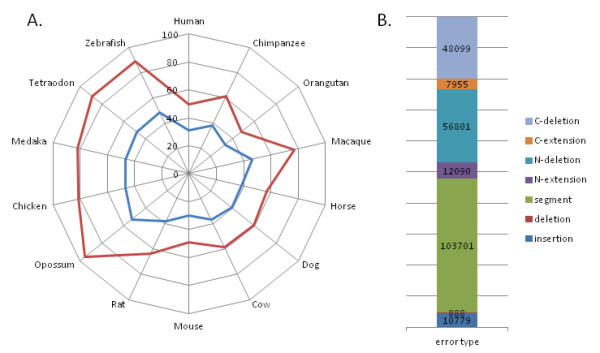
**Estimation of sequence error rates**. A) Percentage of predicted sequence errors in 19,778 protein families in 14 vertebrate genomes. In blue, the percentage of sequences with at least one error. In red, the percentage of total errors observed. B) Classification of sequence errors into 7 types according to their position in the sequence and their nature (see methods). The histogram shows the frequencies of each error type observed in all protein sequences (C-deletion = C-terminal deletion; C-extension = C-terminal extension; N-deletion = N-terminal deletion; N-extension = N-terminal extension; segment = suspicious sequence segment: deletion = internal deletion; insertion = internal insertion).

We predicted protein sequence errors, resulting from genome sequencing errors and exon/intron prediction errors, in the 14 high coverage vertebrate genomes (Table 1) from the Ensembl database, using a previously published method [37]. First, we constructed multiple sequence alignments (MSAs) for each of the 19,778 human protein sequences defined by the Human Proteome Initiative (HPI) and their potential vertebrate homologs. The sequences in the alignments were then clustered into more similar subgroups and errors were predicted if discrepancies were observed between one sequence and its close neighbours, for example between human-chimpanzee or between fish genomes. The error detection protocol was thus used to identify lineage-specific insertions, deletions or sequence segments, which are inconsistent with the conservation information in the MSA. Finally, we calculated the rate of sequence errors found in all 19,778 MSAs (Figure 2A). The MSAs contained a total of 344,437 protein sequences and 240,313 potential sequence errors, giving an estimated sequence error rate of at least 0.7 errors per sequence. The total number of sequences with at least one potential error was 142,836. Thus, on average 41% of sequences were predicted to be erroneous.

**Table 1:** Ensembl genomes used in this study

Genome identifier	Organism	No. of genes	No. of proteins
ENSP	'Human','Homo sapiens'	21971	60953

ENSPTR	'Chimpanzee','Pan troglodytes'	19829	39256

ENSPPY	'Orangutan','Pongo pygmaeus'	20068	29256

ENSMMU	'Macaque','Macaca mulatta'	21905	42370

ENSECA	'Horse','Equus caballus'	20322	28128

ENSCAF	'Dog','Canis familiaris'	19305	29804

ENSBTA	'Cow','Bos taurus'	21036	29517

ENSMUS	'Mouse','Mus musculus'	23873	43630

ENSRNO	'Rat','Rattus norvegicus'	22503	37672

ENSMOD	'Opossum','Monodelphis domestica'	19471	34132

ENSGAL	'Chicken','Gallus gallus'	16736	22945

ENSORL	'Medaka','Oryzias latipes'	19686	25174

ENSTNI	'Tetraodon','Tetraodon nigroviridis'	19602	23909

ENSDAR	'Zebrafish','Danio rerio'	21322	35967

Protein sequences were obtained from the Ensembl database version 51.

The observed error rates were not homogeneous across the different species. Lower rates were observed for the human and mouse proteomes, with 30-31% erroneous sequences, as might be expected for these well studied organisms. Among the non-human primate proteomes considered here, lower error rates were estimated for the orangutan (*Pongo pygmaeus)*, compared to the chimpanzee (*Pan troglodytes*) and especially the Rhesus macaque (*Macaca mulatta)*. The relatively high error rate for the macaque is not surprising since the macaque genome in Ensembl version 51 is a preliminary assembly using whole genome shotgun (WGS) reads from small and medium insert clones. On the other hand, the relative error rates in chimpanzee and orangutan are more surprising. Both the chimpanzee and orangutan genomes have been sequenced to 6x coverage, but in a recent study of primate genome assembly quality, the chimpanzee genome assembly was estimated to be of higher quality [38].

Nevertheless, the same study found that about 70% of inferred errors in the orangutan genome were clustered in the 3.2% of the assembly that is of low quality, implying that > 96% of the assembly could be considered of high fidelity. We found the highest error rates in the opossum, chicken and fish proteomes, with > 45% erroneous sequences. Although these genomes have all been sequenced to high coverage, the lack of a well annotated reference genome from a closely related model organism may result in lower quality protein sequence prediction.

The predicted protein sequence errors were then characterized according to two different factors: (i) the nature of the error, i.e. insertion, deletion or suspicious segment and (ii) the position in the sequence, i.e. at the N/C-terminus or within the sequence. Figure 2B shows the proportion of the different errors observed. The most commonly found error was the presence of a suspicious sequence segment, possibly representing a mispredicted exon. At the N- and C- termini, deletions were observed more frequently than extensions. Although this may be due in part to the protocol used to detect sequence errors, it may also reflect the difficulty of predicting the first and last coding exons. In contrast, internal insertions were more common than internal deletions, suggesting that more internal errors were due to the over-prediction of introns as coding sequences, rather than the under-prediction of exons.

### Comparison of similarity and synteny based homologs

**Figure 3:**
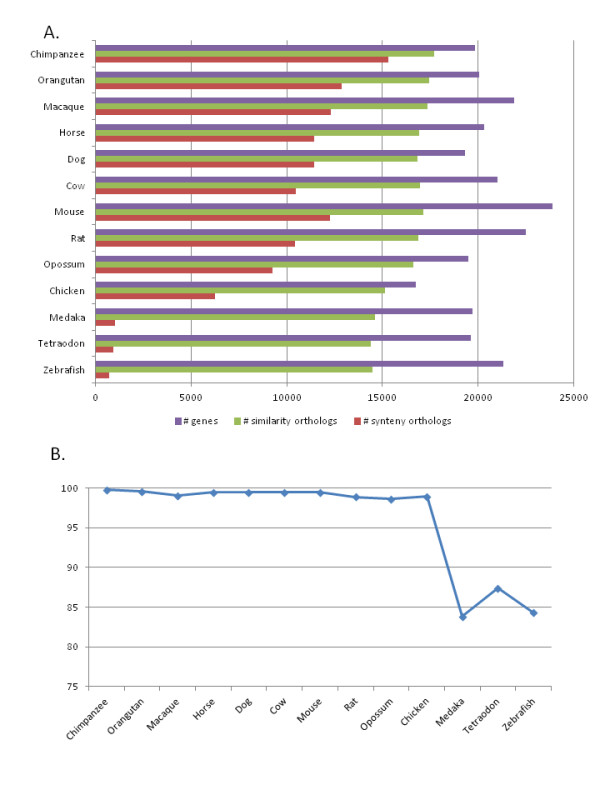
**Number of putative ortholog relationships between human and 13 vertebrate genomes**. A. Putative ortholog relationships between human and each of the 13 vertebrate genomes used in this study were identified by similarity-based and synteny-based approaches. B. The proportion of orthologs predicted by the synteny approach for which the same ortholog was predicted by the similarity-based approach.

Putative orthologs were predicted for each of the 19,778 human proteins based on the MSAs of the human reference sequences and related sequences from the 13 vertebrate genomes. Two different approaches were implemented. First, the sequences from each organism with the smallest evolutionary distance were identified based on pairwise alignments extracted from the MSAs, and denoted "highest similarity homologs". Second, "syntenic homologs" were defined based on the local gene order conservation. The genome coverage achieved by the two methods is shown in Figure 3 and Table S1 in Additional file 1. The highest similarity homologs covered 80% of the 265,658 genes in the 13 vertebrate genomes, ranging from 89% in chimpanzee to 68% in zebrafish. As expected, a smaller proportion (43%) of homologs was found with locally conserved synteny, including 77% of chimpanzee genes and only 3% of zebrafish. Although our definition of locally syntenic regions is relatively stringent, we observe a comparable coverage to other existing methods. For example, we found 51% of mouse genes to be syntenic with human, compared to 59% using the method developed by [39]. Other more refined methods have been developed, such as Syntenator [40], that use less stringent criteria to define conserved syntenic regions. By allowing more gene mismatches and gene insertions/deletions, Syntenator aligned 79% of mouse genes with human.

We then investigated whether the gene that is most similar on the sequence level is also the gene that shares the same gene-neighbourhood (Figure 3 and Table S2 in Additional file 1). Of the 212,409 similarity homologs identified in the 13 vertebrate genomes, 113,517 were found in locally syntenic regions. In mammals, this represents 69% of the highest similarity homologs. This is less than that estimated in a previous study [41], where 97.5% of Inparanoid orthologs in human, mouse, rat and dog were found in syntenic regions, most likely due to our stricter definition of local synteny. On the other hand, only 1% of the identified syntenic homologs (1,157 out of 114,680) were not identified by the similarity-based approach. As expected, a generally higher level of disagreement was observed for more divergent genome pairs. Nevertheless, in human-chicken comparisons, the synteny method identified the same homolog as the similarity approach in 98.8% of the cases. Fewer consistencies were observed in human-fish comparisons (84-87% of syntenic homologs were also the highest similarity homologs), possibly due in part to the whole genome duplications in the fish lineage, resulting in a larger number of paralogs.

### Asymmetric evolution events

We then examined in more detail the 1,157 gene triplets (consisting of the human reference sequence and the two homologs representing putative orthologs in one of the 13 vertebrate genomes), where the syntenic homolog was not the same as the highest similarity homolog. To avoid including chance outcomes caused by very similar rates of sequence evolution of these homologs relative to the human sequence, we identified significantly different rates of evolution at the 95% confidence level (see Methods). Of the 1,157 gene triplets, a total of 688 corresponded to evolutionary scenarios where the syntenic homolog (i.e. the gene copy with the shared genome neighbourhood) evolved significantly faster (Table 2). A complete list of the 688 gene triplets is available in Table S3 in Additional file 1. The alternative scenario for asymmetric evolution where the remote copy evolved faster than the synteny copy is not detected by our protocol. since in this case the homologs defined by similarity and synteny would be the same.

**Table 2:** Number of syntenic homologs with significantly faster evolutionary rates compared to the remote similarity homolog

Genome identifier	No. of syntenic homologs	No. of inconsistencies: syntenic versus highest similarity homologs	Significant asymmetric evolution events (AED)
Human	15295	37	21

Chimpanzee	12881	54	26

Orangutan	12286	121	82

Macaque	11447	59	37

Horse	11443	64	39

Dog	10486	59	30

Cow	12276	70	33

Mouse	10439	117	69

Rat	9261	126	65

Opossum	6231	65	41

Chicken	1027	166	99

Medaka	907	114	83

Tetraodon	701	111	63

Total	114680	1157	688

These may indicate putative asymmetric evolution after duplication (AED) events where the less similar gene copy retained the ancestral gene-neighbourhood.

### Effect of erroneous sequences on prediction of asymmetrical evolution

**Figure 4:**
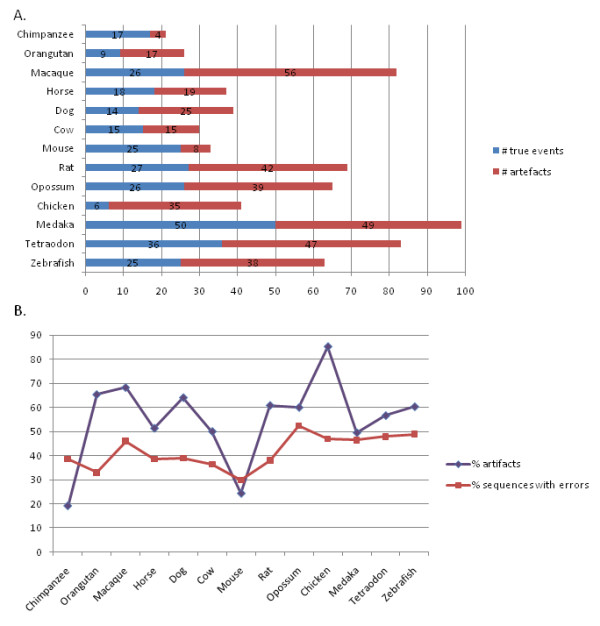
**Effect of erroneous sequences on prediction of asymmetrical evolution in 13 vertebrate genomes**. A. The presence of erroneous sequences give rise to a number of artifactual AED events (shown in red). The remaining events are defined as putative AED events (shown in blue). B. Comparison of percentage of protein sequences predicted to contain errors and percentage of artifactual AED events for each genome.

The 688 gene triplets identified above, consisting of the human reference sequence, the highest similarity homolog and the synteny homolog, constitute a reliable test set representing potential asymmetrical evolution events. To study the impact of errors on the prediction of AED events, we identified erroneous sequences in this test set. Figure 4A shows the number of events that are assumed to be artifacts since at least one of the sequences was predicted to be erroneous, as well as the number of remaining 'true' events. Of the 688 gene triplets, only 294 (43%) do not contain erroneous sequences and may correspond to true events, while a total of 394 (57%) are putative artifacts.

As might be expected, the proportion of artifactual events varies with the different genomes studied, depending on the percentage of erroneous sequence detected (Figure 4B). For example, 19% of chimpanzee and 24% of mouse predicted events are due to artifacts, while this figure increases significantly for the draft macaque and chicken genomes (69% and 88% respectively). It is interesting to note that a larger proportion of artifacts are observed in the orangutan genome than in the chimpanzee, even though the orangutan genome is predicted to contain less sequence errors than the chimpanzee (see above).

In order to validate the putative protein sequence errors leading to artifactual AED events, we investigated the 413 predicted sequence errors in the human reference sequences and their syntenic homologs. The results of the analysis are shown in Table 3 and examples of the different errors detected are provided in Additional file 2. The majority (59%) of the erroneous sequences resulted from DNA sequencing or assembly errors, characterised by the presence of 'N' characters in the DNA sequences. For the remaining 171 protein sequence errors, we searched for the missing protein fragments in the corresponding DNA sequences. For errors involving missing segments (i.e. internal insertion, N/C-terminal extensions or suspicious segments), 89 of the 148 missing segments were detected and we therefore concluded that the error was due to an inaccurate gene structure prediction. In the case of sequence errors corresponding to inserted segments (internal insertions, N/C-terminal insertions), 16 of the 23 inserted segments were conserved in closely related organisms, although 5 of them had one or more stop codons. Finally, we manually verified the transcript evidence in Ensembl for all 23 insertions in gene sequences with no genome errors, as well as for the 59 unconserved deletions. Of these, 62 protein errors were not supported by any transcript information and 9 errors were due to the alternative splicing variants reported for homologous genes. Only 11 (2.7%) of the 413 putative protein sequence errors were identified as false positive predictions, since a transcript was found corresponding to the affected sequence segment.

**Table 3:** Validation of putative protein sequence errors

	Putative protein errors^a^	Genome errors^b^		Exon conservation^c^		Transcript evidence			% FP error^g ^
		**Yes**	**No**	**Yes**	**No**	**No**	**Splicing variants^e^**	**FP error prediction^f^**	

Suspicious segment	223	161	62	43	19	12	3	4	1.8

Deletion	7	1	6	6	0	0	0	0	0.0

N-deletion	68	26	42	19	23	18	2	3	4.4

C-deletion	64	26	38	21	17	16	0	1	1.6

Deletion sub-total	362	214	148	89	59	46	5	8	2.9

	Putative protein errors	Genome errors		Intron conservation^d^		Transcript evidence			% FP error

		Yes	No	Yes (stop)	No	No	Splicing variants	FP error prediction	

Insertion	22	15	7	6 (1)	1	5	2	0	0.0

N-extension	18	7	11	7 (3)	4	7	1	3	16.7

C-extension	11	6	5	3 (1)	2	4	1	0	0.0

Insertion sub-total	51	28	23	16 (5)	7	16	4	3	5.9

Total	413	242	171	100	14	62	9	11	2.7

Putative errors were estimated by analyzing the corresponding gene sequences. ^a^The total number of protein sequence errors included in the analysis. ^b^The number of errors resulting from genome sequencing or assembly errors. ^c^The number of missing segments detected in the corresponding gene sequences. ^d^The number of errors resulting from alternative splicing variants reported for homologous genes. ^e^The number of inserted sequence segments detected in the gene sequences of homologous proteins. The number of these inserted sequence segments with at least one stop codon is given in brackets. ^f^The number of errors supported by transcript evidence, i.e. false positive (FP) error predictions. ^g^The percentage of the total number of putative errors that were invalidated by the analysis.

### Detailed analysis of sequence errors leading to artifactual AED events

**Figure 5:**
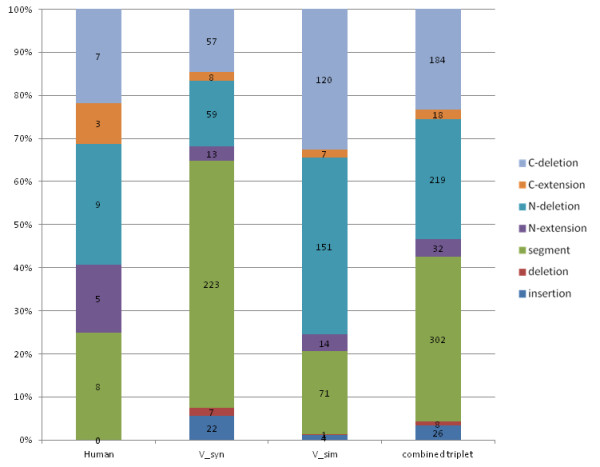
**Characterization of sequence errors in predicted asymmetrical evolution events**. Errors are classified into 7 types according to their position in the sequence and their nature (see methods). The proportions of the different classes found in the human reference sequences, the syntenic homolog (V_syn) and the highest similarity homolog (V_sim) are shown, as well as the proportions observed in the pooled sequences in the gene triplets. (C-deletion = C-terminal deletion; C-extension = C-terminal extension; N-deletion = N-terminal deletion; N-extension = N-terminal extension; segment = suspicious sequence segment: deletion = internal deletion; insertion = internal insertion).

To investigate whether the sequence errors leading to artifactual events were enriched for a particular type, we classified the errors into 7 types as described above. We then calculated the proportion of the different error types found in the gene triplets corresponding to the 688 predicted AED events (Figure 5). In the human reference sequences, only 32 errors were predicted, as might be expected since the human genes have been very widely studied. The majority (24 out of 32) of the human sequence errors were found at the N/C termini, with the exception of a small number of internal sequence segments that were labeled as being suspicious.

When all the sequences in the gene triplets were pooled, no significant enrichment was observed in the frequency distribution of the different error types causing artifactual events, compared to the background distribution observed in all the sequences (as shown in Figure 2). The goodness-of-fit was measured using a likelihood ratio chi-square statistic (chi-square = 3.12, p-value = 0.79). Nevertheless, different error types were observed when the syntenic and highest similarity homologs were considered separately. For example, artifactual events were observed more frequently if the syntenic homolog, i.e. the gene copy that retained the genome neighbourhood after duplication, contained suspicious segments. In contrast, N- and C-deletions in the highest similarity homolog, i.e. the gene copy that was relocated, were more likely to cause artifacts.

**Figure 6:**
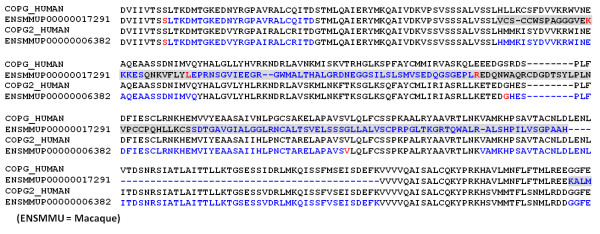
**An example of an artifactual AED event**. Part of the multiple sequence alignment of the human COPG protein sequence [Ensembl:ENSP00000325002] and putative orthologs in the macaque genome. The suspicious segment is boxed in grey. For the Ensembl macaque sequences, exons are colored alternately in black and blue. Residues overlapping splice sites are shown in red.

Figure 6 shows an example of an artifactual event observed in the gene triplet corresponding to [Swiss-prot:COPG_HUMAN] and the two homologs from macaque (the full length alignment is provided in Figure S1 in Additional file 1). The COPG protein forms part of the coatomer complex, involved in protein transport between the endoplasmic reticulum and the Golgi. The macaque syntenic homolog [Ensembl:ENSMMUP00000017291] contains a suspicious segment and an exon deletion that artificially increase its evolutionary distance to human, due to a low quality segment in the genome sequence (indicated by 'N' characters in the gene sequence). Consequently, another macaque protein [Ensembl:ENSMMUP00000006382] is identified as the highest similarity homolog of human COPG, resulting in an artifactual AED event prediction. In fact, [Ensembl:ENSMMUP00000006382] is the ortholog of [Uniprot:COPG2_HUMAN].

The orthology prediction method used in the Ensembl project, based on a phylogenetic gene tree approach, finds the correct 1-to-1 orthology relationship between the human and macaque COPG proteins. Unfortunately, many other orthology databases are less successful. For example, in the Inparanoid database (inparanoid.sbc.su.se), the Ensembl human COPG and macaque COPG2 sequences are in the same orthologous cluster, while no human ortholog is found for the macaque COPG sequence.

### Functional analysis of asymmetrical evolution events

In order to investigate the effect of filtering the erroneous sequences on the subsequent functional analysis of asymmetrical evolution events, we conducted a gene ontology (GO) term enrichment analysis. Specifically, we investigated the 688 AED events detected in this work, where the local syntenic homolog was observed to evolve more rapidly than the relocated highest similarity homolog. At this stage, we excluded 81 events where the human reference sequence had more than one exon, but the relocated homolog had only one exon, since they are likely to be non-functional pseudogenes. For comparison purposes, we used two gene lists: (i) gene list 1 corresponding to the remaining 607 detected events, including both artifactual and putative true events and (ii) gene list 2 corresponding to 250 putative true events only (Table S4 in Additional file 1). The two gene lists were then analyzed for enrichment of GO terms using the AmiGO [42] web server, using the complete set of human genes as the background set and default parameters (Tables S5-6 in Additional file 1). The results of the AmiGO analyses were also submitted to the GO-Module [43] web server, in order to reduce the complexity and identify 'key' GO terms (Table 4).

**Table 4:** GO term enrichment analysis for artifactual and putative AED events

GO enrichment for all events			GO enrichment for true events only		
**GO ID**	**GO biological process**	**P-value**	**GO ID**	**GO biological process**	**P-value**

0032501	multicellular organismal process	4.E-43	0032501	multicellular organismal process	2.E-13

0048856	anatomical structure development	2.E-32	0050896	**response to stimulus**	9.E-12

0065007	biological regulation	4.E-26	0048856	anatomical structure development	3.E-09

0080090	**regulation of primary metabolic process**	6.E-21	0042060	wound healing	2.E-07

0071842	cellular component organization at cellular level	3.E-20	0050789	regulation of biological process	1.E-06

0060255	**regulation of macromolecule metabolic process**	5.E-19	0071842	cellular component organization at cellular level	2.E-06

0051171	**regulation of nitrogen compound metabolic process**	5.E-19	0007596	blood coagulation	4.E-06

0032774	**RNA biosynthetic process**	5.E-16	0022008	**neurogenesis**	5.E-05

2000112	**regulation of cellular macromolecule biosynthetic process**	7.E-16	0006928	**cellular component movement**	6.E-05

0006139	**nucleobase, nucleoside, nucleotide and nucleic acid metabolic process**	1.E-15	0030182	**neuron differentiation**	4.E-04

0010467	**gene expression**	4.E-13			

0042060	wound healing	4.E-09			

0007596	blood coagulation	2.E-08			

0006810	**transport**	2.E-08			

0007166	**cell surface receptor linked signaling pathway**	3.E-06			

0007411	**axon guidance**	5.E-06			

0007601	**visual perception**	2.E-05			

0016477	**cell migration**	5.E-05			

0030168	**platelet activation**	1.E-04			

0006195	**purine nucleotide catabolic process**	1.E-04			

0009207	**purine ribonucleoside triphosphate catabolic process**	5.E-04			

0016568	**chromatin modification**	6.E-04			

0006915	**apoptosis**	8.E-04			

0060173	**limb development**	9.E-04			

Comparison of GO term enrichment analysis for (i) gene list 1 corresponding to 607 predicted asymmetrical evolution events, including both artifactual and putative true events and (ii) 25O true events obtained after filtering the erroneous sequences. GO terms for biological processes were found with P < 10-4 using AmiGO and then filtered with GO-Module (only key terms are shown). Terms that are specific to only one gene list are highlighted in bold.

Gene list 1 was enriched in 24 key GO terms, including a number of vertebrate specializations (e.g. anatomical structure development), but also some fundamental eukaryotic processes (e.g. regulation of metabolic processes, gene expression, axon guidance). For example, the term 'RNA biosynthetic process' is found with a P-value of 5E-16, involving 101 (20%) of the 607 genes in the list. However, only 6 of these 24 key GO terms are associated with the true events in gene list 2. Thus, the remaining 18 (75%) enriched GO terms are probably false positives resulting from the artifactual events. Furthermore, and perhaps more importantly, important key GO terms associated with the true events are not enriched in gene list 1, notably neurogenesis related functions. After filtering of gene triplets with erroneous sequences, gene set 2 was enriched in 10 key terms, including neuron differentiation functions, and response to the environment.

**Figure 7:**
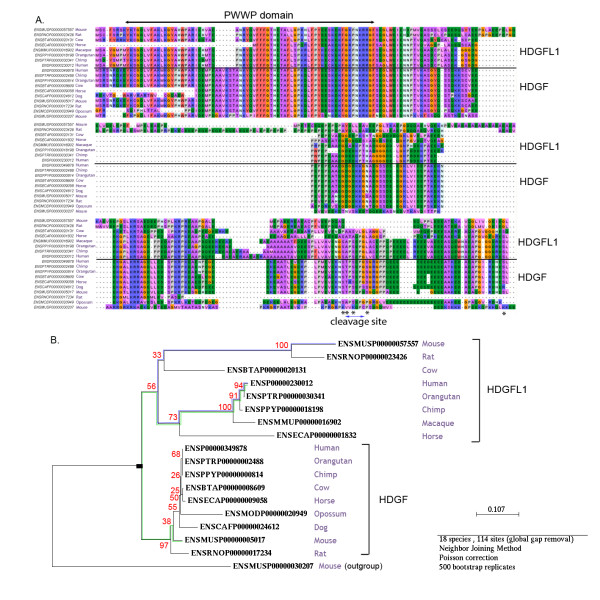
**A putative AED event**. A) Multiple sequence alignment of hepatoma-derived growth factor (HDGF) and HDGF-like proteins. Black lines indicate the two main subgroups corresponding to the duplication node in the phylogenetic tree. Known phosphorylation sites are labeled with asterisks. B) The phylogenetic tree constructed using the Neighbour-Joining algorithm with 500 bootstraps. Bootstrap values for each node are shown in red. The distance between human and mouse HDGF1 sequences (in blue) is longer than the distance between human HDGF1 and mouse HDGF sequences (in green).

Figure 7 shows an example of a true AED event detected in the hepatoma-derived growth factor (HDGF) protein family. The HDGF and HDGF-like family members are characterized by a conserved PWWP domain in the N-terminal region. In human, the HDGF protein [Ensembl:ENSP00000349878] exhibits growth factor properties and has been implicated in organ development and tissue differentiation of the intestine, kidney, liver, and cardiovascular system. In addition, the role of HDGF in cancer biology has recently become a focus of research, since HDGF was found to be over-expressed in a large number of different tumor types (genecards.org). Whereas some family members, such as HDGF and HDGFL2, are expressed in a wide range of tissues, the expression of others is very restricted. For example, HDGFL1 and HDGFL4 are only expressed in testis, although their precise functions are still unknown. We observed an EAD event in several organisms, including mouse and rat. For example, mouse HDGFL1 [Ensembl:ENSMUSP00000057557] on chromosome 13 is syntenic with human HDGFL1 [Ensembl:ENSP00000230012] on chromosome 6, but mouse HDGF [Ensembl:ENSMUSP00000005017] shares higher sequence similarity with human HDGFL1 (58% identity versus 53%). Although mouse HDGFL1 is specifically expressed in testis, like human HDGFL1, the human and mouse proteins are more divergent in the C-terminal region and probably have different functions. In fact, mouse HDGFL1 lacks the caspase cleavage site identified in mouse HDGF, as well as a number of conserved residues that are known to be phosphorylated (genecards.org).

## Discussion

Several recent studies have highlighted the prevalence of errors in genes predicted from genome sequences [24-26,44], particularly in eukaryotic genes. The situation is further complicated by the fact that multiple transcript variants are often expressed by the same gene. Nevertheless, orthology and paralogy, which are fundamental concepts for most evolutionary analyses, are generally defined at the gene level. Many systems, including Ensembl compara [45], simply select the longest transcripts to represent a gene, although there is no guarantee that the longest predicted transcripts in different organisms are equivalent. Some authors have specifically addressed these issues by defining relationships at the transcript level [46,47] or by using processed transcription units, i.e. a combination of all overlapping sequence variants in the genomic region [48]. Nevertheless, these remain partial solutions only and do not resolve all problems.

These quality issues may lead to inaccurate or erroneous conclusions if they are integrated indiscriminately in downstream evolutionary or functional analyses. As an example, when annotating a new genome, gene structure data is often transferred from the genome of a closely related species, e.g., many chimpanzee genes in the Ensembl database were predicted based on comparisons with human transcript data. These gene sequences were then used to perform genome-wide scans for positive selection [49]. Although more positively selected genes were identified in chimpanzees compared to human, it has been suggested that the majority of the signals may be due to errors in the original sequences or in the gene alignments [50]. Thus, we have a vicious circle, where the gene sequences that provide the starting point for most evolutionary analyses are themselves generally predicted based on evolutionary information.

### Protein sequence error rates

We detected erroneous protein sequences based on discrepancies in the conservation of vertebrate protein MSAs. The sequence errors may result from (i) DNA sequencing errors, (ii) badly predicted introns/exons, (iii) different splicing variants predicted in different organisms. We estimated the frequency of erroneous sequences to be at least 41%, although some genomes are more error-prone than others, depending on factors such as sequencing coverage or the availability of a well annotated genome from a closely related organism.

In this study, we only considered sequences from the Ensembl database and we used cross-comparisons between species to identify discrepancies. However, Ensembl may produce predictions that are consistent across organisms, i.e. may reproduce the same errors in different genomes or propagate intron/exon structures. Thus, our estimate of the average sequence error rate is probably conservative. Another recent study [51] showed that the Ensembl compara sequence prediction method correctly identified only 55% of coding transcripts exactly.

### Identification of evolutionary events

Our main goal was to determine to what extent these erroneous sequences affect subsequent evolutionary analyses. We focused on a specific event: gene duplication and the evolutionary fate of paralogs, since gene duplication is often assumed to be the most important source of new functions.

Since duplication events where the local copy has evolved more rapidly may indicate unusual evolutionary scenarios, innovations or adaptations, we specifically searched for examples of such asymmetric evolution events. Our approach involved the identification of reliable AED events that could be used as a test set for estimating the impact of sequence errors. We therefore designed a stringent protocol where we included only high coverage genomes and used the well studied human genome as a reference. We then identified putative orthologs in 13 vertebrate genomes, based on either sequence similarity or local synteny conservation. The similarity-based method used a very simple model of sequence evolution, in order to avoid bias towards one particular model. Nevertheless, this model clearly oversimplifies the complex evolutionary processes involved, and in the future, it would be interesting to investigate the effect of a more realistic model of sequence evolution on AED detection, once sequencing/annotation errors have been removed. We also used a strict definition of local synteny, which led to lower genome coverage in the ortholog prediction step. For the detection of asymmetric evolution, we used a simple measure of amino acid divergence and specified a high significance threshold that would ensure only reliable predictions. Nevertheless, 688 putative AED events were identified that were then used to perform an in-depth investigation of the effect of sequence errors.

### Impact of sequence errors

We compared the syntenic and highest similarity homologs and identified cases where significantly faster evolutionary rates were observed in the syntenic homolog, i.e. the gene copy that retained the genome neighbourhood after duplication, compared to the relocated highest similarity homolog. Initially, 688 AED events were identified, of which 81 similarity homologs were potential retropseudogenes with a reduced exonic map. The majority (57%) of the remaining detected events corresponded to erroneous sequences and only 250 represented putative true AED events. Thus, we conclude that care should be taken when performing genome-wide scans to search for genes with unusual patterns, since outlying genes are more likely to be due to artifacts in the input sequences than the result of true evolutionary events. Furthermore, our in-depth study revealed some of the mechanisms by which errors in the input sequences are propagated during the event prediction. For example, a badly predicted internal segment in one of the homologs results in an increased evolutionary distance to the human reference sequence, while a loss in the more variable N/C-terminal regions artificially reduces the distance. These observations provide guidelines for future error detection and correction strategies that will hopefully allow us to reduce the impact of the sequencing errors.

In asymmetric evolution, one duplicate evolves or degrades faster than the other and often becomes functionally or conditionally specialized. In this context, the accurate detection of the 'functional' homologs, i.e. protein pairs that play functionally equivalent roles [52], is critical. We have shown that orthology assignment and the detection of important genetic events are severely impacted by the high proportion of errors in the initial set of protein sequences, even in high coverage genomes. The errors in the initial data are accumulated and amplified in the higher-level analyses. Our estimated rate of 41% erroneous protein sequences leads to 57% errors in AED event prediction and, in the subsequent Gene Ontology (GO) functional analysis, 75% of the enriched terms are in fact false positives.

The false positive terms in the functional analysis can be very costly to investigate experimentally and a reduction in the false discovery rate is clearly desirable. They are also sufficient to mask some of the true functional enrichments. After filtering the artifactual events corresponding to erroneous sequences, the remaining AED events were enriched in a number of GO categories, including neuron differentiation and response to external stimuli. Interestingly, human-specific duplicates evolving under adaptive natural selection also include genes involved in neuronal and cognitive functions, as well as response to inflammation or stress [53]. Similarly, gene families involved in copy number variations (CNVs) are enriched for similar categories, including interactions with the environment, neurophysiological processes and brain development [54]. A recent study suggested that the relationship between CNVs and positive selection may play an important role in the emergence and evolution of species-specific traits in primates [55]. Genes in many of these categories are thus thought to be important in evolutionary adaptation and to be particular targets of natural selection.

## Conclusions

Up to half of all protein sequences in today's genome databases contain erroneous insertions, deletions or suspicious segments. The high error rates have profound implications, not only for the analysis of protein functions, interaction networks, biochemical pathways or disease phenotypes, but also for our understanding of life's evolution.

The putative sequence errors identified here lead to a significant number of false positives in the detection of asymmetric evolution events, which, if ignored, are sufficient to obscure their true functional significance. We have looked at one important event, asymmetric evolution after duplication, but the effect of protein sequence errors is likely to be similar for other types of events. This might explain many of the contradictions observed in many recent evolutionary studies, aggravating the effects of differences in source data, methodology and planning of experiments [12].

Exploitation of the new genome data is clearly challenging, due to the size of the data sets, their complexity and the high level of noise, and the situation is not likely to improve with low coverage genomes becoming the norm. As a consequence, data cleaning tools and robust statistical analyses will be essential for its reliable interpretation. With as many as 50% erroneous sequences, the simple removal of this data will result in the loss of too much information. It will be necessary to validate and correct the sequence errors and ideally, propagate these corrections to the public databases. Some recent efforts have been undertaken to address these issues [19,26,47], but additional work will be essential to reduce the impact of error and to extract the true meaning hidden in the data.

The alternative is an escalating process where systematic errors are accumulated at each level of the analysis, generating artificially high rates of unusual event predictions and eventually leading to an 'error catastrophe', where the noise overwhelms the true signal.

## Methods

### Protein sequence data sets

Human protein coding genes were retrieved from the Human Proteome Initiative (HPI) and Swiss-prot databases [56], resulting in a total of 19,778 human sequences. Each gene was then used as a query for a BlastP [57] search in a database consisting of the proteomes of 14 vertebrates (Table 1) with almost complete genomes from the Ensembl (version 51) database [36]. The Ensembl human protein sequence with the highest similarity to the HPI query was designated as the reference protein sequence. For each of the 19,778 human reference sequences, potential orthologs were then identified using two different, complementary approaches: sequence similarity and local synteny.

### Putative orthologs based on sequence similarity

For each human reference sequence, a modified version of the PipeAlign [58] protein analysis pipeline was used to construct a multiple sequence alignment (MSA) for all sequences detected by the BlastP search with E < 10^-3 ^(maximum sequences = 500). PipeAlign integrates several steps, including post-processing of the BlastP results, construction of a MSA of the full-length sequences with DbClustal [59], verification of the MSA with RASCAL [60] and removal of unrelated sequences with LEON [61]. In this modified version, DbClustal was replaced by the MAFFT [62] program, since the computational speed of MAFFT is better suited to high throughput projects. The MSAs obtained from this pipeline were then annotated with structural and functional information using MACSIMS [63], an information management system that combines knowledge-based methods with complementary *ab initio *sequence-based predictions. MACSIMS integrates several types of data in the alignment, in particular Gene Ontology annotations, functional annotations and keywords from Swiss-prot, and functional/structural domains from the Pfam database [64].

Based on the MSA, the evolutionary pairwise distance, *d*, between any two sequences was defined as the number of amino acid substitutions per site under the assumption that the number of amino acid substitutions at each site follows the Poisson distribution. Thus:

$$d=-\ln\left(1-p\right)$$

where *d *is the pairwise distance and p is the proportion of different amino acids aligned (dissimilarity).

Then, for each human reference sequence, H_i_, the sequences from the 13 vertebrate organisms with the highest similarity (i.e. the smallest distance) to H_i _were identified and denoted Vn_Sim_i_, where Vn refers to one of the 13 vertebrate organisms (Figure S2A in Additional file 1).

### Putative orthologs based on local synteny

The chromosomal localization of all genes coding for protein sequences was obtained from the Ensembl database. Locally developed software was used to identify regions on the human chromosomes where local synteny was conserved between the human genome and each of the other 13 vertebrate genomes. The chromosomes in each genome are thus represented as a linear sequence of genes. For each human reference sequence, the local syntenic homolog was defined as outlined in (Figure S2B in Additional file 1). For the coding gene, H_i_, at position i on the human genome, its neighbours (H_i-1 _and H_i+1_) were identified. For each of the 13 vertebrate genomes, the sequences with the highest similarity to H_i-1 _and H_i+1 _were selected from the MSA as described above, and denoted Vn_Sim_i-1 _and Vn_Sim_i+1 _respectively, where Vn refers to one of the 13 vertebrate genomes. A local synteny homolog, Vn_Syn_i _exists for H_i _and genome Vn if: (i) homologs were found in Vn for H_i-1 _and H_i+1_, (ii) the separation between the highest similarity homologs, denoted Vn_Sim_i-1 _and Vn_Sim_i+1_, on the genome was less than 5 genes and (iii) a homolog of H_i _was found on the genome between Vn_Sim_i-1 _and Vn_Sim_i+1_. The homolog of H_i _localized between V_Sim_i-1 _and V_Sim_i+1 _with the highest similarity (smallest evolutionary distance) to the human reference sequence was then defined as the syntenic homolog.

Genes with ambiguous genomic locations, such as scaffolds etc., were discarded since the synteny relationship could not be reliably established. In addition, local or tandem duplications were excluded since the genome contexts of the two gene copies were similar. Although tandem duplicates should be adjacent to each other on one chromosome, extensive gene inversions may insert irrelevant genes into the tandem arrays. We therefore used a stringent threshold and excluded cases where Vn_Sim_i _and Vn_Syn_i _were separated on the genomes by less than 10 genes.

### Automatic detection of potential sequence errors

**Figure 8:**
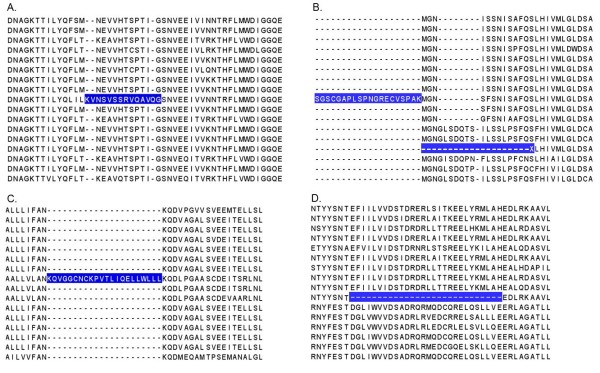
**Detection of potential sequence errors**. Examples of sequence discrepancies (highlighted in blue) that are identified in the subfamily alignments. A) Potential mispredicted exons, resulting in suspicious sequence segments, are identified based on the conserved blocks in the subfamily alignment. B) Potential start and stop site errors are predicted based on the distribution of the positions of the N/C-terminal residues. C) Identification of a potential inserted intron, based on the presence of a single sequence with the insertion in a given subfamily. D) Identification of a potential missing exon, based on the presence of a single sequence with a deletion in a given subfamily.

For each MSA corresponding to a human reference sequence, an automatic protocol was used to detect sequence discrepancies that may indicate gene prediction errors. Different types of prediction error were considered, such as excluding coding exons, including introns as part of the coding sequence, or wrongly predicting start and termination sites. The protocol is described in detail elsewhere [37]. Briefly, the sequences in the MSA were first clustered into more related subfamilies then, for each subfamily, sequences with potential errors were identified using an empirical rule-based approach. (i) Badly predicted exons were identified using the RASCAL algorithm [60] as outliers or 'suspicious' sequence segments (Figure 8A). (ii) Badly predicted start or stop sites were identified by considering the positions of the N/C-terminal residues for each sequence in the subfamily alignment (Figure 8B). Normal values were defined as lying within the lower and upper quartiles of the distribution of terminal positions. Sequences with terminal positions outside this window were annotated as potential deletion/extension errors. (iii) Inserted introns (Figure 8C) were detected if a single sequence contained an insertion of more than 10 residues. (iv) Missing exons (Figure 8D) were detected if a single sequence contained a deletion of more than 10 residues.

Each error was then classified in one of 7 different classes: internal insertions, internal deletions, suspicious sequence segments, extensions at the N- or C-terminus, and deletions at the N- or C-terminus.

### Validation of potential sequence errors

The errors in the protein sequences were estimated by analysing the corresponding DNA gene sequences from the Ensembl database. First, if the gene sequence contained a run of 'N' characters, we assumed that the predicted protein sequence error was the result of a DNA sequencing or assembly error. Second, the gene sequences with no 'N' characters were searched for the missing protein sequence fragments. For errors corresponding to internal deletions, deletions at the N- or C- terminus or suspicious sequence segments, the missing protein fragment was extracted from a closely related sequence in the multiple alignment. The protein fragment was then aligned to the gene sequence from the ENSEMBL database using the PairWise software [65]. The fragment was considered to be present in the gene sequence if the percent identity of the protein and translated gene sequences was greater than a given threshold. The threshold used here was specific to the pair of organisms compared and was defined as the lower quartile of the protein sequence identities for the complete proteomes of the two organisms. A similar protocol was used for the errors corresponding to insertions in a given protein sequence, except that, in this case, the protein fragment corresponding to the insertion was aligned to the gene sequence of another closely related sequence. Finally, the transcript evidence for the protein sequences in the Ensembl database was searched manually for known transcripts and splicing variants.

### Prediction of asymmetrical evolutionary rates

It has been suggested that, after a gene duplication event, one duplicate generally maintains the ancestral function while the other is free to evolve and acquire novel functionality. This scenario implies that the protein with conserved functionality will undergo less sequence evolution than the one exploring new functionalities. To determine which of the two homologs described above (highest sequence similarity or syntenic) was more likely to share the same function as the human reference sequence, we estimated the difference between the two evolutionary distances: human reference to similarity homolog and human reference to syntenic homolog. Thus, for each of the 13 vertebrate genomes considered in this study, we have a triplet of homologs, H_i_, Vn_sim_i_, Vn_syn_i_, and we want to estimate the difference Δ between two distances *d(H_i_, Vn_sim_i_) *and *d(H_i_, Vn_syn_i_)*.

We used an estimator based on pairwise sequence distances similar to one defined previously, that is relatively fast to compute and has almost the same statistical power as the widely used maximum likelihood estimator [66]. The distance, *d*, between two sequences is defined as the number of amino acid substitutions per site under the assumption that the number of amino acid substitutions at each site follows the Poisson distribution, as before. The variance σ of the distance *d *is given by:

$$\sigma^{2}\left(d\right)=p∕\left[\left(1-p\right)n\right]$$

where *p *is the proportion of amino acid differences and *n *is the total number of amino acids compared.

If X has two homologs Y and Z, and Y is the closest homolog to X, an estimator for the difference in evolutionary distances is:

$$\Delta =d\left(X,Y\right)-d\left(X,Z\right)$$

The variance of the difference can be computed as:

$$\begin{array}{ll} \sigma^{2}\left(\Delta \right) & =\sigma^{2}\left(d\left(X,Y\right)\right)+\sigma^{2}\left(d\left(X,Z\right)\right)\;\\ & \;-2cov\left(d\left(X,Y\right),d\left(X,Z\right)\right)\;\\ & \; \end{array}$$

and thus, an upper bound for the variance of the estimator is:

$$\sigma^{2}\left(\Delta \right)=\sigma^{2}\left(d\left(X,Y\right)\right)+\sigma^{2}\left(d\left(X,Z\right)\right)$$

Finally, we assume X,Y are significantly closer than X,Z if:

$$\Delta <-\text{k}.\sigma \left(\Delta \right)$$

In this work, the parameter *k *was set to 1.96, reflecting the 95% confidence level. Thus, we would expect 5% of the tested gene triplets to falsely reject the hypothesis of asymmetrical evolution.

## Authors' contributions

FP participated in the design of the study, constructed the multiple alignments and synteny data, and helped draft the manuscript. BL designed and carried out the ortholog predictions and participated in the analysis of the data. PP participated in the design of the study and the genetic event analysis, and helped draft the manuscript. OP participated in the design and coordination of the study and the analysis of the data and helped draft the manuscript. JDT conceived the study, participated in its design and coordination, and helped to analyse the data and to draft the manuscript. All authors read and approved the final manuscript.

## Supplementary Material

Additional file 1 **Supporting figures and tables**. Supporting figures and tables for the manuscript are provided as a PDF file. Click here for file

Additional file 2 **Examples of erroneous protein sequences and their validation**. Example text and figures are provided as a PDF file. Click here for file
