# Supplementary material for: Controversies in modern evolutionary biology: the imperative for error detection and quality control
Source: BMC Genomics. 2012 Jan 4;13:5. doi: 10.1186/1471-2164-13-5 (PMC3311146; doi:10.1186/1471-2164-13-5)
Supplement: Additional file 1 — Supporting figures and tables. Supporting figures and tables for the manuscript are provided as a PDF file. [file 1471-2164-13-5-S1.PDF]

Figure S1. Full length alignment of sequences in figure 7.

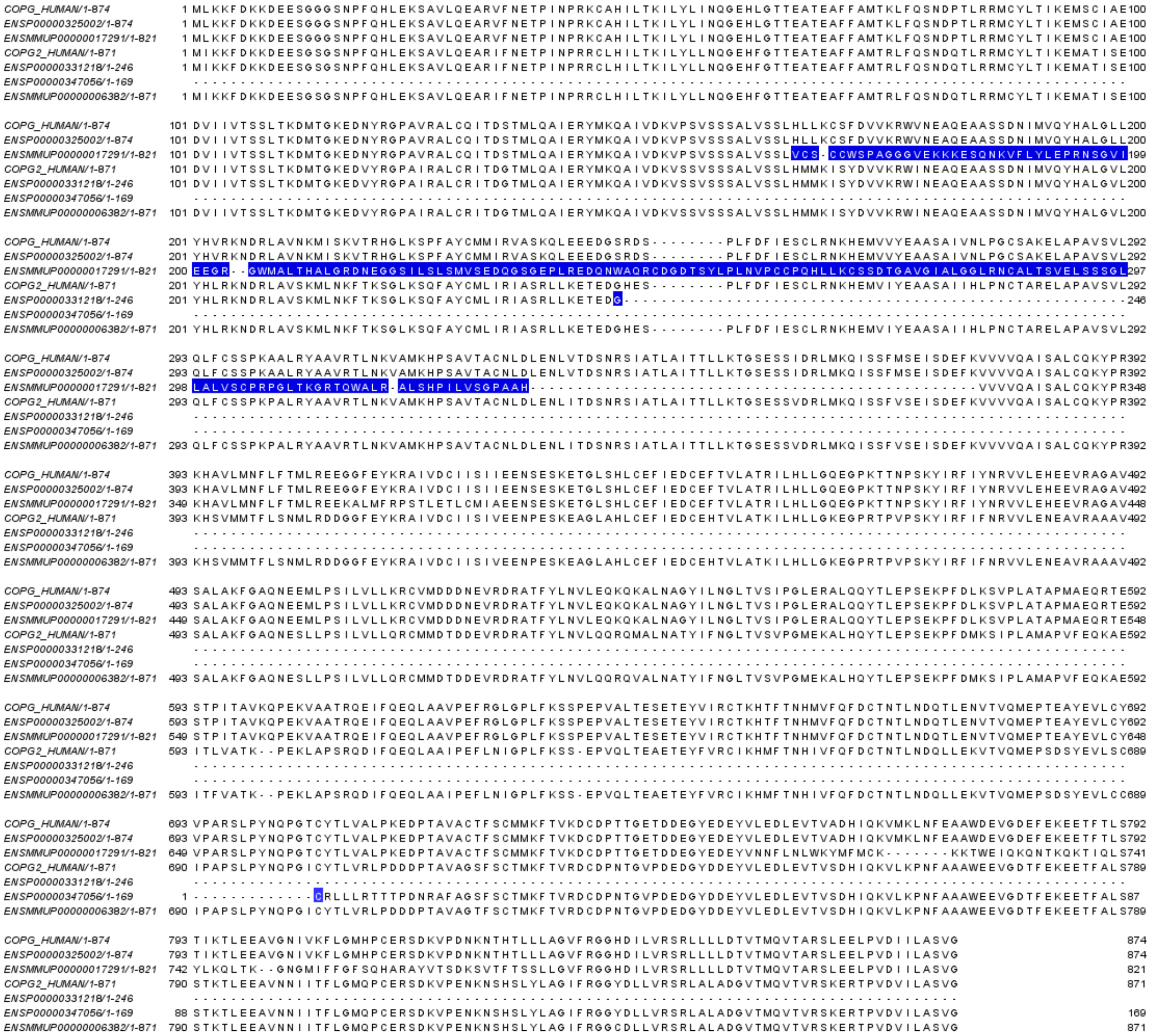

Figure S2. Identification of potential orthologs based on either sequence similarity or local synteny.

A) Schematic view of a multiple sequence alignment (MSA) containing the human reference sequence Hi and homologs from 3 different vertebrate genomes V1-V3. For each vertebrate genome Vn, a similarity-based ortholog for Hi, Vn_Simi is defined as the sequence with the smallest evolutionary distance to Hi. The other sequences in the MSA, labeled H_Paraj or Vn_Paraj, are assumed to be paralogs. B) Schematic view of conserved gene order between the human genome and another vertebrate genome. For each vertebrate genome Vn, a synteny-based ortholog for Hi, Vn_Syni, is defined if similarity orthologs exist for Hi-1, Hi+1 and Vn_Syni is homologous to Hi, and the separation between these orthologs on the genome is less than 5 genes (orange box indicates an insertion of one gene in the vertebrate genome).

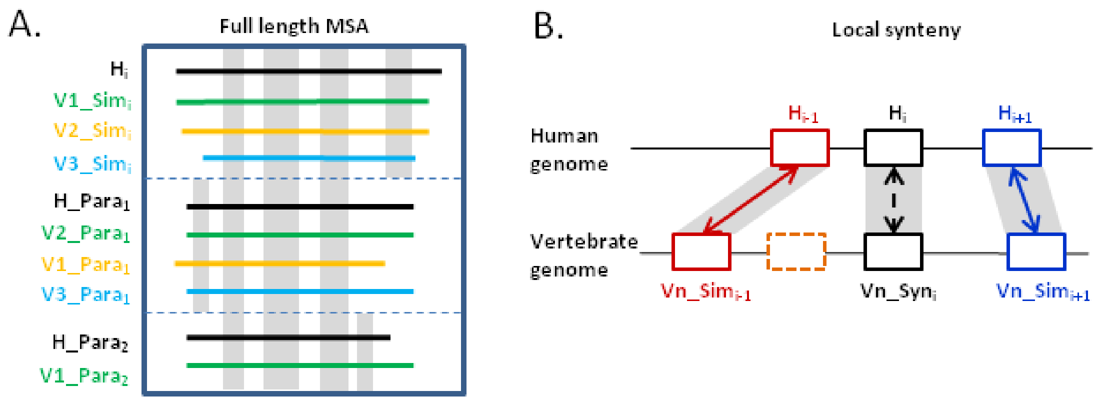

Table S1. Number of putative orthologs identified by similarity-based and synteny-based approaches

Genome identifier	No. genes (Ensembl 51)	No. similarity orthologs	No. synteny orthologs	% similarity orthologs	% synteny orthologs
ENSPTR	19829	17700	15295	89	77
ENSPPY	20068	17448	12881	87	64
ENSMMU	21905	17343	12286	79	56
ENSECA	20322	16914	11447	83	56
ENSCAF	19305	16851	11443	87	59
ENSBTA	21036	16949	10486	81	50
ENSMUS	23873	17118	12276	72	51
ENSRNO	22503	16894	10439	75	46
ENSMOD	19471	16600	9261	85	48
ENSGAL	16736	15147	6231	91	37
ENSORL	19686	14601	1027	74	5
ENSTNI	19602	14374	907	73	5
ENSDAR	21322	14470	701	68	3
Total	265658	212409	114680	80	43

Table S2. Consistency between ortholog sets predicted by similarity and synteny based approaches.

	Overlap between synteny and similarity methods	% of similarity orthologs	% of synteny orthologs
ENSPTR	15258	86.2	99.8
ENSPPY	12827	73.5	99.6
ENSMMU	12165	70.1	99.0
ENSECA	11388	67.3	99.5
ENSCAF	11379	67.5	99.4
ENSBTA	10427	61.5	99.4
ENSMUS	12206	71.3	99.4
ENSRNO	10322	61.1	98.9
ENSMOD	9135	55.0	98.6
ENSGAL	6166	40.7	99.0
ENSORL	861	5.9	83.8
ENSTNI	793	5.5	87.4
ENSDAR	590	4.1	84.2
total	113517	53.4	99.0

Table S3. 688 detected asymmetric evolution events, where the ortholog in the ancestral genome neighborhood has evolved faster than the distant ortholog.

Human reference protein	Chr	Vertebrate synteny ortholog	Chr	%ID	Vertebrate similarity ortholog	Chr	%ID
ENSP00000355023	X	ENSECAP00000012471	X	64.4	ENSECAP00000004101	24	72.7
ENSP00000367913	X	ENSRNOP00000055260	X	37.7	ENSRNOP00000057472	14	46.8
ENSP00000343671	X	ENSPPYP00000022655	X	95.3	ENSPPYP00000022404	Un	96.5
ENSP00000343671	X	ENSDARP00000091915	9	39.5	ENSDARP00000083040	11	49
ENSP00000367705	X	ENSORLP00000014631	21	32.2	ENSORLP00000001675	10	38.3
ENSP00000382840	X	ENSDARP00000002260	9	81.5	ENSDARP00000010461	6	83.5
ENSP00000367284	X	ENSMMUP00000004669	X	88.4	ENSMMUP00000004448	6	97.4
ENSP00000343244	X	ENSBTAP00000002365	X	65.1	ENSBTAP00000023205	17	69.7
ENSP00000364126	X	ENSRNOP00000000179	X	65.4	ENSRNOP00000037807	14	73.6
ENSP00000364107	X	ENSMMUP00000023726	X	81.9	ENSMMUP00000003292	1	86.2
ENSP00000364054	X	ENSECAP00000002255	X	74.8	ENSECAP00000022481	23	83.5
ENSP00000329115	X	ENSMUSP00000026601	X	41.7	ENSMUSP00000108170	X	54.2
ENSP00000329115	X	ENSRNOP00000034830	X	39.3	ENSRNOP00000049196	X	44.9
ENSP00000355119	X	ENSMUSP00000073522	X	41	ENSMUSP00000073074	2	50.6
ENSP00000361998	X	ENSMUSP00000108849	X	57	ENSMUSP00000065645	18	61.2
ENSP00000361998	X	ENSRNOP00000048993	X	48.4	ENSRNOP00000029450	7	63.2
ENSP00000361917	X	ENSECAP00000001022	X	33.5	ENSECAP00000009285	4	51.9
ENSP00000361859	X	ENSMMUP00000040914	X	91.3	ENSMMUP00000004663	X	93.4
ENSP00000339068	X	ENSPPYP00000023056	X	92.3	ENSPPYP00000020680	8	96.7
ENSP00000361544	X	ENSMUSP00000098772	X	45.6	ENSMUSP00000056785	7	53.6
ENSP00000361544	X	ENSRNOP00000045126	X	46.3	ENSRNOP00000027278	1	53.6
ENSP00000303777	X	ENSCAFP00000026589	X	48.3	ENSCAFP00000030721	X	64.8
ENSP00000200639	X	ENSORLP00000001324	10	33.9	ENSORLP00000015872	21	39.4
ENSP00000200639	X	ENSDARP00000018069	14	31.9	ENSDARP00000027333	9	41.6
ENSP00000360367	X	ENSMODP00000012972	X	85.6	ENSMODP00000037142	5	95
ENSP00000360165	X	ENSMUSP00000062570	X	70.9	ENSMUSP00000050377	4	74.2
ENSP00000360090	X	ENSRNOP00000006726	X	68.9	ENSRNOP00000043128	9	74.6
ENSP00000218364	X	ENSMODP00000017614	X	64.4	ENSMODP00000000232	1	67.6
ENSP00000243314	X	ENSCAFP00000028200	X	46.3	ENSCAFP00000033026	X	54.6
ENSP00000218316	X	ENSGALP00000014739	4	43.9	ENSGALP00000027791	1	47
ENSP00000218316	X	ENSMODP00000007121	X	42.5	ENSMODP00000006150	5	47.4
ENSP00000383493	c6	ENSPTRP00000055488	6	67.5	ENSPTRP00000030539	6	94.3
ENSP00000372713	c6	ENSPPYP00000019310	6	53.8	ENSPPYP00000018450	6	97.6
ENSP00000349016	1	ENSMMUP00000026273	1	91.5	ENSMMUP00000034214	6	99.3
ENSP00000365435	1	ENSCAFP00000024172	2	68.3	ENSCAFP00000022550	9	73.9
ENSP00000328915	1	ENSPTRP00000040832	1	58.9	ENSPTRP00000052839	1	97.5
ENSP00000365343	1	ENSPTRP00000000336	1	95.6	ENSPTRP00000000337	Un	96.9
ENSP00000365266	1	ENSMUSP00000043718	4	35.5	ENSMUSP00000102760	4	49.1
ENSP00000365266	1	ENSRNOP00000033783	5	35.5	ENSRNOP00000041054	14	45.3
ENSP00000235799	1	ENSMODP00000003109	4	33.9	ENSMODP00000001670	2	37.6

ENSP00000364524	1	ENSPPYP00000002090	1	99.5	ENSPPYP00000014773	2b	100
ENSP00000364219	1	ENSDARP00000068888	6	66.2	ENSDARP00000035352	2	76.9
ENSP00000326261	1	ENSCAFP00000019053	2	70.3	ENSCAFP00000035033	25	77.7
ENSP00000326261	1	ENSBTAP00000040625	2	67.3	ENSBTAP00000022329	1	86.8
ENSP00000270879	1	ENSMODP00000018103	4	44.9	ENSMODP00000029388	1	51.9
ENSP00000257070	1	ENSMODP00000021907	4	41	ENSMODP00000023210	2	54.1
ENSP00000381316	1	ENSDARP00000052168	19	18.2	ENSDARP00000063483	22	22.7
ENSP00000362566	1	ENSECAP00000013902	2	85	ENSECAP00000004344	15	93.8
ENSP00000362208	1	ENSMMUP00000026892	1	82.6	ENSMMUP00000017787	2	86.8
ENSP00000362110	1	ENSMODP00000022109	4	81.9	ENSMODP00000032714	8	91.4
ENSP00000361373	1	ENSGALP00000032535	8	23.1	ENSGALP00000036579	4	37.5
ENSP00000361095	1	ENSTNIP00000009744	15	37.6	ENSTNIP00000005902	1	47.6
ENSP00000361095	1	ENSORLP00000013274	17	32.7	ENSORLP00000014149	4	48
ENSP00000309565	1	ENSMMUP00000005620	1	72.4	ENSMMUP00000034975	5	97.8
ENSP00000360958	1	ENSPPYP00000001605	1	95	ENSPPYP00000011941	1	96.9
ENSP00000383836	1	ENSMMUP00000005820	1	33.7	ENSMMUP00000003965	4	37.8
ENSP00000385967	1	ENSMMUP00000004853	1	95.9	ENSMMUP00000011565	1	98.7
ENSP00000360269	1	ENSGALP00000017645	8	43.6	ENSGALP00000014571	3	48.9
ENSP00000294428	1	ENSORLP00000012628	4	44.7	ENSORLP00000006870	8	48.8
ENSP00000359799	1	ENSMMUP00000039878	1	60.7	ENSMMUP00000003886	Un	100
ENSP00000359146	1	ENSPPYP00000001293	1	99.2	ENSPPYP00000011876	1	100
ENSP00000358888	1	ENSDARP00000072115	11	42.4	ENSDARP00000001793	8	47.8
ENSP00000314520	1	ENSORLP00000010656	7	86.7	ENSORLP00000008061	5	90.7
ENSP00000348786	1	ENSMODP00000001599	2	93	ENSMODP00000009084	8	95.6
ENSP00000358646	1	ENSMMUP00000020569	1	59.8	ENSMMUP00000017010	2	88.7
ENSP00000358414	1	ENSGALP00000004672	8	64.7	ENSGALP00000023936	Z	67.1
ENSP00000343749	1	ENSPPYP00000001095	1	59.2	ENSPPYP00000011937	1	81.7
ENSP00000358010	1	ENSMMUP00000016840	1	70	ENSMMUP00000010938	7	75.7
ENSP00000311083	1	ENSMMUP00000008659	1	92.5	ENSMMUP00000036256	17	97.5
ENSP00000323424	1	ENSRNOP00000015471	2	75.9	ENSRNOP00000045693	X	81.1
ENSP00000305347	1	ENSMMUP00000011782	1	85	ENSMMUP00000029366	Un	87.7
ENSP00000357127	1	ENSCAFP00000034756	38	79.4	ENSCAFP00000035448	Un	84.1
ENSP00000357086	1	ENSMUSP00000091861	1	24	ENSMUSP00000036380	1	37.3
ENSP00000357086	1	ENSRNOP00000000059	13	25.9	ENSRNOP00000047320	2	37.9
ENSP00000357040	1	ENSCAFP00000018506	38	51.7	ENSCAFP00000014338	17	73.7
ENSP00000356995	1	ENSMMUP00000038560	1	93.1	ENSMMUP00000035504	Un	100
ENSP00000356885	1	ENSMODP00000001428	2	87.8	ENSMODP00000033263	2	92.8
ENSP00000239462	1	ENSDARP00000071646	2	48.7	ENSDARP00000005621	5	54.3
ENSP00000356591	1	ENSMODP00000007588	2	74	ENSMODP00000018793	1	76
ENSP00000356587	1	ENSTNIP00000002910	1	35.7	ENSTNIP00000007930	4	44.7
ENSP00000294742	1	ENSMMUP00000037912	1	67.8	ENSMMUP00000027157	15	73.5
ENSP00000356438	1	ENSDARP00000090671	2	68.5	ENSDARP00000003684	20	73.3
ENSP00000314299	1	ENSPTRP00000003010	1	86.3	ENSPTRP00000044519	1	96.1
ENSP00000356319	1	ENSGALP00000003403	8	14.5	ENSGALP00000017285	3	38.9
ENSP00000330460	1	ENSPPYP00000023464	1	98	ENSPPYP00000011895	1	100

ENSP00000356237	1	ENSECAP00000008886	30	70.3	ENSECAP00000010875	28	83.4
ENSP00000241651	1	ENSGALP00000000227	26	72.2	ENSGALP00000037631	1	81.2
ENSP00000347041	1	ENSORLP00000019181	23	45.8	ENSORLP00000019666	5	50.6
ENSP00000355924	1	ENSTNIP00000003462	14	58.8	ENSTNIP00000020841	5	70
ENSP00000355924	1	ENSORLP00000018714	24	63.2	ENSORLP00000013764	3	74.5
ENSP00000340900	1	ENSTNIP00000000073	14	25	ENSTNIP00000019989	10	27.5
ENSP00000355675	1	ENSGALP00000038397	2	58.3	ENSGALP00000018300	7	66.7
ENSP00000355636	1	ENSRNOP00000024491	19	93.5	ENSRNOP00000055446	X	95.1
ENSP00000262861	1	ENSMMUP00000015832	1	97.7	ENSMMUP00000032235	Un	100
ENSP00000264181	1	ENSMMUP00000018736	1	93	ENSMMUP00000018737	Un	99
ENSP00000318650	1	ENSDARP00000072950	17	62.7	ENSDARP00000008325	12	70.5
ENSP00000349785	1	ENSMMUP00000037496	1	90.5	ENSMMUP00000020886	16	93.9
ENSP00000355460	1	ENSMMUP00000005833	1	73.7	ENSMMUP00000035484	Un	83.7
ENSP00000342818	1	ENSMUSP00000104688	2	50.5	ENSMUSP00000089089	9	57.1
ENSP00000334794	1	ENSMMUP00000037562	1	54.1	ENSMMUP00000032249	Un	92.2
ENSP00000355435	1	ENSMMUP00000037562	1	71.9	ENSMMUP00000037471	Un	90.4
ENSP00000324687	1	ENSPTRP00000050949	1	56.2	ENSPTRP00000003749	1	94.4
ENSP00000384219	2	ENSTNIP00000011126	10	70.2	ENSTNIP00000013634	14	87.2
ENSP00000384219	2	ENSDARP00000047088	17	13.2	ENSDARP00000044396	20	87.4
ENSP00000256707	2	ENSORLP00000018861	22	69.8	ENSORLP00000022722	24	75.9
ENSP00000352904	2	ENSDARP00000020092	13	64.6	ENSDARP00000039965	20	69.7
ENSP00000175091	2	ENSMODP00000021278	4	30.5	ENSMODP00000012268	1	83.3
ENSP00000295148	2	ENSMODP00000038218	Un	52.9	ENSMODP00000011745	1	55.6
ENSP00000386082	2	ENSDARP00000095268	17	74.6	ENSDARP00000091534	20	81.1
ENSP00000233121	2	ENSTNIP00000022333	14	69	ENSTNIP00000022494	10	78.9
ENSP00000282412	2	ENSTNIP00000010277	17	74.1	ENSTNIP00000008010	2	78.5
ENSP00000282412	2	ENSDARP00000011295	13	76.7	ENSDARP00000011459	12	83.2
ENSP00000385991	2	ENSGALP00000012796	3	17.6	ENSGALP00000018146	5	78.1
ENSP00000384974	2	ENSDARP00000022350	13	99.4	ENSDARP00000091642	1	100
ENSP00000234396	2	ENSECAP00000020289	15	83.8	ENSECAP00000014612	2	89
ENSP00000264447	2	ENSMODP00000028155	1	67.2	ENSMODP00000034037	8	77.4
ENSP00000377617	2	ENSMMUP00000012744	13	71.2	ENSMMUP00000014938	2	90
ENSP00000374788	2	ENSMMUP00000036337	13	90.2	ENSMMUP00000006018	13	95.9
ENSP00000381065	2	ENSRNOP00000056552	9	75.6	ENSRNOP00000051629	1	100
ENSP00000264258	2	ENSMMUP00000012930	13	95	ENSMMUP00000012975	Un	99.2
ENSP00000302756	2	ENSTNIP00000020747	12	44.5	ENSTNIP00000001693	7	56.9
ENSP00000263239	2	ENSBTAP00000042872	2	89.8	ENSBTAP00000048691	4	96.3
ENSP00000365477	2	ENSECAP00000009906	18	89.8	ENSECAP00000005898	18	94.4
ENSP00000295206	2	ENSDARP00000023487	9	52.8	ENSDARP00000094379	1	62.6
ENSP00000318197	2	ENSPPYP00000014962	2b	97.8	ENSPPYP00000005899	13	99.1
ENSP00000301920	2	ENSTNIP00000013266	3	50.7	ENSTNIP00000009491	2	54.4
ENSP00000364664	2	ENSTNIP00000019597	2	78	ENSTNIP00000016267	3	79.8
ENSP00000376478	2	ENSTNIP00000019604	2	42.2	ENSTNIP00000016261	3	53.5
ENSP00000263817	2	ENSORLP00000021369	21	66.6	ENSORLP00000004294	2	70.1
ENSP00000284669	2	ENSTNIP00000020040	2	60	ENSTNIP00000016249	3	62.6

ENSP00000234160	2	ENSTNIP00000020049	2	59.6	ENSTNIP00000010714	15	70.6
ENSP00000376379	2	ENSTNIP00000020051	2	42.1	ENSTNIP00000014674	3	47
ENSP00000376379	2	ENSORLP00000021635	21	41.3	ENSORLP00000004128	8	47.7
ENSP00000263812	2	ENSORLP00000021697	21	73.6	ENSORLP00000017936	17	78.6
ENSP00000264106	2	ENSORLP00000021757	21	51.6	ENSORLP00000018008	17	54.4
ENSP00000380262	2	ENSORLP00000021911	21	63.9	ENSORLP00000008036	2	83.3
ENSP00000272748	2	ENSDARP00000087622	9	54.7	ENSDARP00000011283	6	58.6
ENSP00000376322	2	ENSDARP00000076768	9	38.1	ENSDARP00000052661	16	51.5
ENSP00000249504	2	ENSDARP00000075052	9	58.4	ENSDARP00000067694	11	90
ENSP00000338225	2	ENSORLP00000013194	21	54.1	ENSORLP00000020591	16	57.1
ENSP00000384159	2	ENSMODP00000035308	4	82.6	ENSMODP00000025143	2	86
ENSP00000260950	2	ENSORLP00000018869	21	61.5	ENSORLP00000018605	5	64.8
ENSP00000305675	2	ENSDARP00000092325	9	48.2	ENSDARP00000095865	6	61.3
ENSP00000376115	2	ENSORLP00000006086	11	58.2	ENSORLP00000020658	16	65.6
ENSP00000286195	2	ENSMODP00000019010	7	21.1	ENSMODP00000019039	1	46.8
ENSP00000295414	2	ENSMMUP00000026606	12	84.2	ENSMMUP00000003647	19	88.3
ENSP00000352709	2	ENSRNOP00000033369	9	83.5	ENSRNOP00000043624	5	98.9
ENSP00000379890	2	ENSTNIP00000017321	16	59.7	ENSTNIP00000009139	1	72
ENSP00000302251	2	ENSRNOP00000026419	9	97.7	ENSRNOP00000017854	4	100
ENSP00000339115	2	ENSORLP00000001365	4	44.9	ENSORLP00000005339	7	49.6
ENSP00000287766	3	ENSORLP00000005782	5	76.3	ENSORLP00000014556	7	81.6
ENSP00000273038	3	ENSORLP00000005637	5	56.5	ENSORLP00000016518	9	62.1
ENSP00000285094	3	ENSORLP00000006086	11	66.2	ENSORLP00000020658	16	78.8
ENSP00000341024	3	ENSDARP00000056204	19	50.6	ENSDARP00000083224	16	73.6
ENSP00000379854	3	ENSMMUP00000000072	2	89.3	ENSMMUP00000019538	14	94.3
ENSP00000283627	3	ENSMMUP00000038308	2	53.2	ENSMMUP00000022239	Un	98.5
ENSP00000307599	3	ENSGALP00000038088	2	57.5	ENSGALP00000017797	7	62.8
ENSP00000232974	3	ENSGALP00000008464	2	77.7	ENSGALP00000039496	27	86.2
ENSP00000296130	3	ENSORLP00000002778	11	49.7	ENSORLP00000005719	16	56.2
ENSP00000307156	3	ENSGALP00000035462	12	60.5	ENSGALP00000038566	1	67.4
ENSP00000265538	3	ENSPPYP00000015522	3	81.8	ENSPPYP00000011947	1	98.5
ENSP00000232496	3	ENSDARP00000096613	22	66.1	ENSDARP00000069296	4	71.6
ENSP00000294189	3	ENSMUSP00000080203	9	82.9	ENSMUSP00000108123	12	94.4
ENSP00000294189	3	ENSRNOP00000014849	8	83.1	ENSRNOP00000045151	19	88.2
ENSP00000295899	3	ENSBTAP00000043944	22	98.5	ENSBTAP00000012008	17	100
ENSP00000295569	3	ENSTNIP00000014063	11	64.1	ENSTNIP00000018093	9	79.2
ENSP00000182096	3	ENSMODP00000021223	7	66.9	ENSMODP00000003367	4	75.8
ENSP00000373194	3	ENSMUSP00000078873	16	76.8	ENSMUSP00000073737	16	81
ENSP00000296161	3	ENSGALP00000019701	7	37.5	ENSGALP00000004449	27	41.3
ENSP00000325002	3	ENSMMUP00000017291	11	74.9	ENSMMUP00000006382	3	81.1
ENSP00000376930	3	ENSTNIP00000021084	9	78.6	ENSTNIP00000017706	11	82
ENSP00000309762	3	ENSPTRP00000026513	3	98.2	ENSPTRP00000035127	8	100
ENSP00000310785	3	ENSPPYP00000015834	3	95.8	ENSPPYP00000010163	18	97.2
ENSP00000329419	3	ENSORLP00000019501	13	74.4	ENSORLP00000017646	17	90.3
ENSP00000232217	3	ENSDARP00000048837	2	67.9	ENSDARP00000093236	15	75.4

ENSP00000308361	3	ENSORLP00000000420	13	34.1	ENSORLP00000007515	13	43.7
ENSP00000319096	3	ENSORLP00000007391	13	91.8	ENSORLP00000019850	17	94
ENSP00000374287	3	ENSDARP00000059393	18	77.9	ENSDARP00000080552	11	85.1
ENSP00000231706	3	ENSORLP00000003666	20	65	ENSORLP00000014368	22	74.2
ENSP00000263967	3	ENSRNOP00000014527	2	94.5	ENSRNOP00000037275	X	97.8
ENSP00000232564	3	ENSRNOP00000014871	2	77.3	ENSRNOP00000022649	5	90.9
ENSP00000372005	3	ENSRNOP00000016179	2	51.9	ENSRNOP00000012530	15	58.3
ENSP00000265022	3	ENSMODP00000016623	2	63	ENSMODP00000017825	8	80.4
ENSP00000341031	3	ENSORLP00000019542	4	35.1	ENSORLP00000005615	7	42
ENSP00000327197	3	ENSRNOP00000035285	11	83.8	ENSRNOP00000056000	2	98.2
ENSP00000326806	3	ENSMODP00000019849	7	88.5	ENSMODP00000029243	2	91.7
ENSP00000383007	4	ENSORLP00000000360	10	45.2	ENSORLP00000002647	7	52.6
ENSP00000339381	4	ENSORLP00000007588	18	45.9	ENSORLP00000018188	1	56.8
ENSP00000281228	4	ENSPTRP00000053732	4	90.2	ENSPTRP00000027412	Un	93.5
ENSP00000317445	4	ENSCAFP00000032489	3	38.3	ENSCAFP00000021252	4	45.9
ENSP00000385347	4	ENSTNIP00000016024	20	71.6	ENSTNIP00000006927	18	78.3
ENSP00000306772	4	ENSDARP00000043555	1	71.2	ENSDARP00000054371	15	93.2
ENSP00000346408	4	ENSMMUP00000035387	5	68.2	ENSMMUP00000033731	6	77.7
ENSP00000353129	4	ENSCAFP00000002672	13	77.7	ENSCAFP00000002669	6	98.2
ENSP00000305964	4	ENSMUSP00000060890	5	58.8	ENSMUSP00000033582	X	70
ENSP00000305964	4	ENSRNOP00000056258	14	62.3	ENSRNOP00000054370	X	70
ENSP00000295454	4	ENSGALP00000022923	4	68.2	ENSGALP00000026978	1	81.3
ENSP00000329803	4	ENSRNOP00000032032	14	55.2	ENSRNOP00000058140	12	69.1
ENSP00000336752	4	ENSDARP00000063608	20	53	ENSDARP00000094123	1	65.9
ENSP00000342181	4	ENSRNOP00000002862	14	88	ENSRNOP00000048248	1	93.4
ENSP00000311816	4	ENSCAFP00000003276	13	83.1	ENSCAFP00000022285	3	90.7
ENSP00000251566	4	ENSBTAP00000025005	6	61.1	ENSBTAP00000051040	6	74.2
ENSP00000264908	4	ENSDARP00000013086	5	51.4	ENSDARP00000096118	14	66.7
ENSP00000351706	4	ENSRNOP00000045775	14	83	ENSRNOP00000047647	X	88.4
ENSP00000295470	4	ENSMODP00000014933	5	93.8	ENSMODP00000008450	6	95.9
ENSP00000302216	4	ENSDARP00000072108	8	54.8	ENSDARP00000057865	12	68.8
ENSP00000295266	4	ENSMUSP00000060774	3	74.7	ENSMUSP00000033662	X	86
ENSP00000295266	4	ENSRNOP00000021719	2	75.3	ENSRNOP00000030279	X	86
ENSP00000265149	4	ENSRNOP00000016446	2	61.3	ENSRNOP00000054510	4	71.1
ENSP00000343885	4	ENSORLP00000008244	1	58.9	ENSORLP00000008491	15	61.9
ENSP00000378163	4	ENSRNOP00000009046	2	86.2	ENSRNOP00000054703	17	98.3
ENSP00000304736	4	ENSMODP00000038335	5	70.7	ENSMODP00000016038	2	95.8
ENSP00000347004	4	ENSMODP00000007179	5	56.2	ENSMODP00000017945	1	66.3
ENSP00000283131	4	ENSRNOP00000024568	19	97.9	ENSRNOP00000029841	17	99.7
ENSP00000336923	4	ENSTNIP00000020881	18	44.9	ENSTNIP00000021374	7	53.3
ENSP00000309982	4	ENSBTAP00000006095	17	32.7	ENSBTAP00000049751	22	43.9
ENSP00000368046	4	ENSMMUP00000002781	5	81	ENSMMUP00000002782	7	89.6
ENSP00000231357	5	ENSGALP00000036193	2	40.4	ENSGALP00000030349	11	94
ENSP00000339283	5	ENSORLP00000003460	12	55.4	ENSORLP00000002985	4	64.6
ENSP00000339283	5	ENSDARP00000015197	10	57.1	ENSDARP00000046669	11	61.4

ENSP00000274361	5	ENSMODP00000002464	3	27.1	ENSMODP00000025251	3	32.2
ENSP00000321326	5	ENSTNIP00000002202	4	41.2	ENSTNIP00000018495	12	50.2
ENSP00000342940	5	ENSTNIP00000016470	4	72	ENSTNIP00000018449	12	76.6
ENSP00000342940	5	ENSORLP00000002962	12	68.8	ENSORLP00000004062	9	76.1
ENSP00000339730	5	ENSTNIP00000018477	12	61.9	ENSTNIP00000012955	1	71.5
ENSP00000339730	5	ENSORLP00000011233	9	63.9	ENSORLP00000006242	12	88.7
ENSP00000339730	5	ENSDARP00000051004	5	66.2	ENSDARP00000070464	20	72.6
ENSP00000369486	5	ENSORLP00000012403	9	54	ENSORLP00000001255	12	79.1
ENSP00000325819	5	ENSECAP00000016214	14	82.9	ENSECAP00000006362	1	95.9
ENSP00000326540	5	ENSMODP00000032048	1	69.6	ENSMODP00000037389	5	74.1
ENSP00000231487	5	ENSMMUP00000017138	6	88.8	ENSMMUP00000041158	18	100
ENSP00000304669	5	ENSBTAP00000018861	7	98.7	ENSBTAP00000018863	7	99.7
ENSP00000302543	5	ENSTNIP00000000372	1	36.9	ENSTNIP00000021202	18	41.7
ENSP00000239451	5	ENSMUSP00000052849	18	78.9	ENSMUSP00000033871	8	85
ENSP00000239451	5	ENSRNOP00000027128	18	75.5	ENSRNOP00000016043	16	84.7
ENSP00000378005	5	ENSTNIP00000021373	7	50.4	ENSTNIP00000011799	1	56.5
ENSP00000349459	5	ENSGALP00000005544	13	20.2	ENSGALP00000034658	Z	36.2
ENSP00000367139	5	ENSMUSP00000062976	18	23.2	ENSMUSP00000069247	18	38.3
ENSP00000231656	5	ENSGALP00000039659	13	76.4	ENSGALP00000038682	4	90
ENSP00000296951	5	ENSTNIP00000020197	1	57	ENSTNIP00000003786	7	59.2
ENSP00000322898	5	ENSTNIP00000019239	7	90.4	ENSTNIP00000020195	1	94.4
ENSP00000377547	5	ENSCAFP00000025446	4	85.7	ENSCAFP00000013853	32	88.9
ENSP00000296930	5	ENSMMUP00000020738	6	93.5	ENSMMUP00000030737	11	99.6
ENSP00000296930	5	ENSCAFP00000024874	4	91.8	ENSCAFP00000008796	17	97.8
ENSP00000265100	5	ENSBTAP00000020092	20	97.2	ENSBTAP00000016378	19	98.6
ENSP00000265100	5	ENSMODP00000031188	1	95.2	ENSMODP00000009945	2	98.6
ENSP00000253490	5	ENSPTRP00000029981	5	93.8	ENSPTRP00000040688	5	95.5
ENSP00000323714	5	ENSMUSP00000097103	13	56.6	ENSMUSP00000029803	3	63.7
ENSP00000323714	5	ENSMODP00000006273	1	73.7	ENSMODP00000021132	7	78.9
ENSP00000348099	5	ENSCAFP00000024059	4	84.4	ENSCAFP00000030959	X	90.2
ENSP00000264870	6	ENSDARP00000018091	24	42.7	ENSDARP00000053566	7	48.8
ENSP00000341680	6	ENSDARP00000094783	19	61.5	ENSDARP00000071887	16	65.3
ENSP00000368341	6	ENSGALP00000020723	2	68.4	ENSGALP00000038189	20	78.9
ENSP00000244776	6	ENSMMUP00000025990	4	95.9	ENSMMUP00000025537	9	100
ENSP00000230012	6	ENSMUSP00000057557	13	52.6	ENSMUSP00000005017	3	58.5
ENSP00000230012	6	ENSRNOP00000023426	17	55.3	ENSRNOP00000017234	2	64.8
ENSP00000230012	6	ENSECAP00000001832	20	48.3	ENSECAP00000009058	5	58.1
ENSP00000297012	6	ENSMUSP00000072227	13	88.3	ENSMUSP00000088288	3	94.6
ENSP00000297012	6	ENSRNOP00000039289	17	90.7	ENSRNOP00000040177	2	94.6
ENSP00000350580	6	ENSBTAP00000036521	23	98.5	ENSBTAP00000053002	3	100
ENSP00000307705	6	ENSRNOP00000022735	17	57.1	ENSRNOP00000024304	17	85.5
ENSP00000366378	6	ENSBTAP00000051771	23	54.4	ENSBTAP00000051036	23	88.5
ENSP00000259953	6	ENSMMUP00000034158	4	96.9	ENSMMUP00000039140	Un	99.1
ENSP00000347271	6	ENSRNOP00000000582	20	87	ENSRNOP00000046992	10	97
ENSP00000244458	6	ENSTNIP00000021476	11	68.4	ENSTNIP00000015546	9	73.9

ENSP00000363149	6	ENSTNIP00000002369	9	62.5	ENSTNIP00000021475	11	74.1
ENSP00000362973	6	ENSMMUP00000023964	4	97.8	ENSMMUP00000040467	Un	99.3
ENSP00000361962	6	ENSMMUP00000028077	4	97.6	ENSMMUP00000012698	7	99.2
ENSP00000185206	6	ENSBTAP00000013537	23	71.7	ENSBTAP00000048950	2	76.5
ENSP00000381654	6	ENSTNIP00000019668	17	62.9	ENSTNIP00000000883	10	65.7
ENSP00000358497	6	ENSMMUP00000026456	4	99.8	ENSMMUP00000036360	Un	100
ENSP00000358358	6	ENSORLP00000022430	24	45.7	ENSORLP00000013911	22	70
ENSP00000357973	6	ENSDARP00000062977	20	54.8	ENSDARP00000023654	17	67.2
ENSP00000357840	6	ENSMODP00000029578	2	64.6	ENSMODP00000010832	Un	67.1
ENSP00000357621	6	ENSMMUP00000011716	4	93.4	ENSMMUP00000040450	1	94.9
ENSP00000357440	6	ENSORLP00000019407	24	54.5	ENSORLP00000017265	6	62
ENSP00000275200	6	ENSDARP00000068971	20	32.8	ENSDARP00000091247	20	40.3
ENSP00000351206	6	ENSDARP00000017195	20	44.9	ENSDARP00000094225	19	60.1
ENSP00000326624	6	ENSBTAP00000022407	9	8.2	ENSBTAP00000008495	1	15.9
ENSP00000376112	6	ENSTNIP00000015218	14	76.2	ENSTNIP00000022511	10	78.1
ENSP00000356320	6	ENSRNOP00000030143	1	20.5	ENSRNOP00000004694	13	25.3
ENSP00000356202	6	ENSMMUP00000025081	4	89.5	ENSMMUP00000018725	X	93.5
ENSP00000327315	6	ENSTNIP00000020001	10	55.3	ENSTNIP00000005069	14	59.8
ENSP00000384196	6	ENSECAP00000002730	31	95.2	ENSECAP00000003082	9	97.4
ENSP00000356015	6	ENSMMUP00000016874	4	88.8	ENSMMUP00000016873	5	90.7
ENSP00000354590	6	ENSMUSP00000095028	17	82.4	ENSMUSP00000090857	11	91.2
ENSP00000326348	7	ENSPPYP00000019392	7	55.2	ENSPPYP00000013182	22	64.4
ENSP00000329902	7	ENSECAP00000018615	13	15.8	ENSECAP00000019054	11	36.5
ENSP00000258738	7	ENSGALP00000017711	2	53.5	ENSGALP00000014477	2	58
ENSP00000222718	7	ENSDARP00000074915	19	30	ENSDARP00000023072	16	72.1
ENSP00000353151	7	ENSECAP00000000275	4	34.1	ENSECAP00000003063	6	52.9
ENSP00000006015	7	ENSECAP00000010944	4	85.4	ENSECAP00000002054	18	90.7
ENSP00000265395	7	ENSTNIP00000012066	21	65.6	ENSTNIP00000021598	8	77.1
ENSP00000379612	7	ENSMODP00000032334	8	80.8	ENSMODP00000026427	X	82.9
ENSP00000283928	7	ENSORLP00000009424	16	80.7	ENSORLP00000006437	11	88.2
ENSP00000344536	7	ENSRNOP00000017713	17	55	ENSRNOP00000006095	7	61.2
ENSP00000318158	7	ENSPPYP00000019732	7	85.7	ENSPPYP00000022429	Un	98.9
ENSP00000258775	7	ENSMUSP00000049490	11	53.5	ENSMUSP00000073532	10	64
ENSP00000258775	7	ENSRNOP00000047425	14	53.8	ENSRNOP00000003550	7	64
ENSP00000258775	7	ENSMODP00000033236	6	28	ENSMODP00000024778	Un	68.2
ENSP00000313050	7	ENSBTAP00000042249	25	80.8	ENSBTAP00000048210	10	85.5
ENSP00000252037	7	ENSPPYP00000019622	7	88.9	ENSPPYP00000020481	7	95.4
ENSP00000306330	7	ENSTNIP00000001215	7	93.9	ENSTNIP00000019330	16	96.8
ENSP00000322885	7	ENSRNOP00000001950	12	85.8	ENSRNOP00000045042	X	90.6
ENSP00000265362	7	ENSORLP00000011874	6	73.5	ENSORLP00000018857	23	75.8
ENSP00000344831	7	ENSGALP00000030590	2	59.8	ENSGALP00000039598	27	67.1
ENSP00000344831	7	ENSORLP00000011718	16	56.5	ENSORLP00000001859	8	60.8
ENSP00000369419	7	ENSMODP00000013015	8	76.9	ENSMODP00000017660	1	80.3
ENSP00000005260	7	ENSORLP00000011904	8	54.1	ENSORLP00000004171	19	56.4
ENSP00000292475	7	ENSMMUP00000025227	3	95.7	ENSMMUP00000038266	12	97.9

ENSP00000329565	7	ENSPTRP00000047793	7	92.2	ENSPTRP00000047749	7	96.5
ENSP00000344242	7	ENSTNIP00000015085	13	42.6	ENSTNIP00000021671	19	49.4
ENSP00000385374	7	ENSMMUP00000005914	3	72.2	ENSMMUP00000001909	2	76.1
ENSP00000385374	7	ENSCAFP00000035307	18	64.5	ENSCAFP00000008183	20	77.3
ENSP00000265441	7	ENSTNIP00000019151	13	67.4	ENSTNIP00000009835	11	71.7
ENSP00000377047	7	ENSCAFP00000005230	14	58.6	ENSCAFP00000010664	20	69.5
ENSP00000386148	7	ENSCAFP00000034261	16	84.8	ENSCAFP00000034963	Un	87.3
ENSP00000386148	7	ENSECAP00000002543	4	74.2	ENSECAP00000005535	4	80.6
ENSP00000339867	7	ENSORLP00000015139	16	65	ENSORLP00000007710	11	67.1
ENSP00000297537	7	ENSORLP00000021913	17	67.9	ENSORLP00000001702	2	91.5
ENSP00000262177	7	ENSCAFP00000007728	16	77.9	ENSCAFP00000007729	12	84.9
ENSP00000262177	7	ENSMODP00000006478	8	78.8	ENSMODP00000029650	2	83.3
ENSP00000334330	8	ENSMUSP00000076067	8	58.1	ENSMUSP00000060899	8	66.7
ENSP00000355279	8	ENSRNOP00000016882	16	99.6	ENSRNOP00000057288	16	100
ENSP00000289963	8	ENSRNOP00000012993	15	82.3	ENSRNOP00000052349	2	86.5
ENSP00000385026	8	ENSDARP00000075037	17	72.9	ENSDARP00000094564	20	77
ENSP00000306999	8	ENSTNIP00000022362	14	41.6	ENSTNIP00000020266	6	50.4
ENSP00000344667	8	ENSECAP00000015839	27	19.2	ENSECAP00000019157	3	40.8
ENSP00000321445	8	ENSORLP00000010073	20	58.4	ENSORLP00000020645	24	64
ENSP00000379497	8	ENSORLP00000011160	16	53.4	ENSORLP00000006814	3	59.6
ENSP00000379278	8	ENSORLP00000003299	16	79.5	ENSORLP00000012885	7	87.3
ENSP00000251808	8	ENSORLP00000003257	16	55.4	ENSORLP00000003636	11	62.8
ENSP00000321507	8	ENSDARP00000069192	16	53.3	ENSDARP00000052078	19	55.8
ENSP00000371740	9	ENSPPYP00000021572	9	97.4	ENSPPYP00000022286	9	99.4
ENSP00000371463	9	ENSRNOP00000034834	1	88.7	ENSRNOP00000053338	X	95
ENSP00000369757	9	ENSBTAP00000049139	8	89.2	ENSBTAP00000006990	2	98.8
ENSP00000369562	9	ENSPPYP00000021489	9	81.5	ENSPPYP00000022287	9	94.1
ENSP00000369127	9	ENSECAP00000008597	23	99.2	ENSECAP00000002642	13	100
ENSP00000350844	9	ENSTNIP00000007750	18	77.1	ENSTNIP00000016123	1	81
ENSP00000366161	9	ENSDARP00000019037	5	66.5	ENSDARP00000022273	10	71.3
ENSP00000365926	9	ENSMMUP00000029000	15	72.7	ENSMMUP00000019468	X	92.7
ENSP00000365908	9	ENSGALP00000032176	Z	29.9	ENSGALP00000002798	28	56.6
ENSP00000365908	9	ENSMODP00000034920	6	54.9	ENSMODP00000000835	3	60.9
ENSP00000297814	9	ENSTNIP00000008747	12	37	ENSTNIP00000012160	5	43.5
ENSP00000317788	9	ENSMODP00000010448	6	99.1	ENSMODP00000023704	5	99.8
ENSP00000364995	9	ENSGALP00000017365	Z	67.7	ENSGALP00000011095	25	72.3
ENSP00000364711	9	ENSDARP00000064800	22	52.3	ENSDARP00000048890	23	63
ENSP00000364398	9	ENSTNIP00000000397	12	41.6	ENSTNIP00000009737	15	44.6
ENSP00000363939	9	ENSMUSP00000029991	4	80.6	ENSMUSP00000099944	11	84.5
ENSP00000311679	9	ENSMUSP00000103178	4	39	ENSMUSP00000086191	17	44.3
ENSP00000311679	9	ENSRNOP00000020207	5	39	ENSRNOP00000039434	1	44.6
ENSP00000363373	9	ENSGALP00000036830	Z	67	ENSGALP00000003035	28	78.2
ENSP00000363360	9	ENSMODP00000005949	6	91.3	ENSMODP00000005968	3	99
ENSP00000348385	9	ENSCAFP00000004927	11	44.4	ENSCAFP00000031041	6	46.4
ENSP00000377854	9	ENSORLP00000012486	12	69.6	ENSORLP00000000611	9	76.7

ENSP00000297913	9	ENSCAFP00000030735	9	47.4	ENSCAFP00000033821	20	54.9
ENSP00000362717	9	ENSDARP00000055328	21	60.2	ENSDARP00000029550	8	85.6
ENSP00000362588	9	ENSDARP00000021834	5	92.6	ENSDARP00000048993	8	96.9
ENSP00000354772	9	ENSORLP00000016634	9	56.2	ENSORLP00000016554	12	73.8
ENSP00000354812	9	ENSMMUP00000039950	15	54.7	ENSMMUP00000039680	Un	100
ENSP00000361453	9	ENSTNIP00000011875	4	65.2	ENSTNIP00000008581	12	68.9
ENSP00000361453	9	ENSORLP00000008914	12	63.7	ENSORLP00000021982	9	69.4
ENSP00000263610	9	ENSDARP00000089097	5	80.1	ENSDARP00000016114	21	82.5
ENSP00000361101	9	ENSMMUP00000001702	15	88.1	ENSMMUP00000023552	15	93.2
ENSP00000263604	9	ENSMMUP00000028316	15	93.9	ENSMMUP00000035305	Un	99
ENSP00000346797	9	ENSMODP00000021905	1	52.7	ENSMODP00000029090	2	66.5
ENSP00000341289	9	ENSCAFP00000028782	9	97.7	ENSCAFP00000027491	20	98.6
ENSP00000350954	10	ENSORLP00000006281	20	80	ENSORLP00000007775	17	84.8
ENSP00000315299	10	ENSCAFP00000006379	2	80.3	ENSCAFP00000025799	8	84.6
ENSP00000315299	10	ENSECAP00000004472	29	79.9	ENSECAP00000006446	15	84.6
ENSP00000367952	10	ENSDARP00000030232	4	43.9	ENSDARP00000026630	25	50.7
ENSP00000298943	10	ENSECAP00000019343	29	71.8	ENSECAP00000003777	18	89.8
ENSP00000366801	10	ENSGALP00000014088	2	55.2	ENSGALP00000019107	7	69.9
ENSP00000320025	10	ENSORLP00000013669	20	74.1	ENSORLP00000008202	2	76.2
ENSP00000365811	10	ENSMUSP00000092751	2	91.5	ENSMUSP00000023468	16	96.2
ENSP00000318602	10	ENSORLP00000016386	20	59.1	ENSORLP00000012441	10	67.9
ENSP00000346986	10	ENSCAFP00000005880	2	95.8	ENSCAFP00000005870	2	97.4
ENSP00000363921	10	ENSORLP00000005962	20	61.6	ENSORLP00000002648	17	65.7
ENSP00000379054	10	ENSECAP00000002802	1	66.6	ENSECAP00000015292	3	70.4
ENSP00000362975	10	ENSORLP00000006728	15	29.5	ENSORLP00000009165	1	32.4
ENSP00000362849	10	ENSBTAP00000047226	28	58.3	ENSBTAP00000018863	7	63
ENSP00000265865	10	ENSPPYP00000002782	10	26.8	ENSPPYP00000018036	5	57.7
ENSP00000362336	10	ENSORLP00000008519	15	82.3	ENSORLP00000001135	14	89.9
ENSP00000211998	10	ENSORLP00000011805	15	81.7	ENSORLP00000010700	19	87.6
ENSP00000311051	10	ENSTNIP00000016148	2	47.5	ENSTNIP00000019649	17	58.8
ENSP00000361686	10	ENSTNIP00000016146	2	76.2	ENSTNIP00000011469	17	82.7
ENSP00000361616	10	ENSTNIP00000011470	17	43.3	ENSTNIP00000016145	2	49.3
ENSP00000361435	10	ENSPPYP00000002679	10	99.2	ENSPPYP00000006043	13	100
ENSP00000361435	10	ENSCAFP00000022943	4	98.5	ENSCAFP00000022283	Un	100
ENSP00000361177	10	ENSDARP00000021117	13	50.1	ENSDARP00000069322	12	55.1
ENSP00000355296	10	ENSORLP00000003266	15	52.8	ENSORLP00000013126	19	59.1
ENSP00000344658	10	ENSPTRP00000004729	10	48.3	ENSPTRP00000027970	4	59.8
ENSP00000360802	10	ENSGALP00000010434	6	10.4	ENSGALP00000039113	13	52.4
ENSP00000337500	10	ENSECAP00000013804	1	95.7	ENSECAP00000011696	17	97.1
ENSP00000265997	10	ENSORLP00000000895	15	78.1	ENSORLP00000002130	14	81
ENSP00000360502	10	ENSTNIP00000022094	2	68.3	ENSTNIP00000011502	17	71.3
ENSP00000359634	10	ENSORLP00000009559	15	73.2	ENSORLP00000014372	1	81.3
ENSP00000354616	10	ENSORLP00000010541	15	88.5	ENSORLP00000015489	1	93.9
ENSP00000359022	10	ENSGALP00000008983	6	41.6	ENSGALP00000036878	4	50.7
ENSP00000358311	10	ENSBTAP00000026896	26	74.3	ENSBTAP00000042381	6	88.3

ENSP00000357949	10	ENSCAFP00000018502	28	85.9	ENSCAFP00000018542	Un	90.8
ENSP00000357881	10	ENSORLP00000011937	19	55	ENSORLP00000007006	15	71.1
ENSP00000339850	10	ENSMODP00000038202	1	54.3	ENSMODP00000020822	1	75.7
ENSP00000382707	11	ENSMMUP00000017430	14	94.7	ENSMMUP00000032893	7	97
ENSP00000332788	11	ENSMMUP00000037878	14	77.8	ENSMMUP00000040405	Un	92.8
ENSP00000322724	11	ENSMUSP00000065147	7	76.5	ENSMUSP00000095823	7	82.7
ENSP00000322593	11	ENSMODP00000027676	4	58.4	ENSMODP00000013954	4	62.6
ENSP00000347997	11	ENSCAFP00000033488	21	30.1	ENSCAFP00000002072	1	51.8
ENSP00000341581	11	ENSMUSP00000102472	7	55.3	ENSMUSP00000102489	7	61.6
ENSP00000341581	11	ENSRNOP00000023007	1	54.3	ENSRNOP00000021468	1	61.9
ENSP00000323224	11	ENSRNOP00000050895	1	66.8	ENSRNOP00000049559	1	88.5
ENSP00000344984	11	ENSPPYP00000004050	11	16.1	ENSPPYP00000019730	7	27.4
ENSP00000299459	11	ENSECAP00000003406	7	83	ENSECAP00000001291	7	85.1
ENSP00000308820	11	ENSORLP00000008603	3	40.7	ENSORLP00000001439	6	47.2
ENSP00000340281	11	ENSTNIP00000012231	5	71.9	ENSTNIP00000016721	13	83.6
ENSP00000355332	11	ENSTNIP00000000026	13	80.7	ENSTNIP00000012228	5	90
ENSP00000324948	11	ENSORLP00000001551	6	60.6	ENSORLP00000008419	3	65.7
ENSP00000347883	11	ENSTNIP00000007569	13	46.4	ENSTNIP00000012253	5	56.5
ENSP00000347883	11	ENSORLP00000001572	6	51.4	ENSORLP00000008406	3	57.7
ENSP00000280706	11	ENSMODP00000008066	5	69.4	ENSMODP00000024452	1	73.8
ENSP00000298937	11	ENSRNOP00000006388	3	67.1	ENSRNOP00000021604	2	79.3
ENSP00000278385	11	ENSTNIP00000004019	13	20.1	ENSTNIP00000014489	2	24.1
ENSP00000321419	11	ENSMODP00000024885	5	57.6	ENSMODP00000024909	5	70.1
ENSP00000329056	11	ENSPTRP00000006258	11	59.7	ENSPTRP00000006254	11	83.5
ENSP00000324831	11	ENSMODP00000024909	5	56.5	ENSMODP00000024930	5	69.7
ENSP00000323853	11	ENSECAP00000004896	12	63.5	ENSECAP00000005325	12	83.1
ENSP00000279783	11	ENSECAP00000004896	12	84.3	ENSECAP00000003696	12	86.6
ENSP00000310117	11	ENSMMUP00000037437	14	58.2	ENSMMUP00000006532	8	97.3
ENSP00000310117	11	ENSRNOP00000040750	1	96.6	ENSRNOP00000028724	17	98.8
ENSP00000301896	11	ENSMMUP00000037490	14	53.9	ENSMMUP00000041118	Un	97.1
ENSP00000310620	11	ENSBTAP00000051015	29	61.6	ENSBTAP00000024786	21	73.5
ENSP00000353701	11	ENSRNOP00000026968	1	84.6	ENSRNOP00000007288	3	93.4
ENSP00000317469	11	ENSMMUP00000014613	14	95	ENSMMUP00000014612	Un	97.2
ENSP00000365936	11	ENSMODP00000009512	8	87.7	ENSMODP00000014419	3	91.2
ENSP00000324960	11	ENSMODP00000009288	8	61.1	ENSMODP00000026669	Un	68.5
ENSP00000294304	11	ENSMMUP00000037689	14	94.6	ENSMMUP00000012039	Un	99.3
ENSP00000263309	11	ENSMODP00000006180	4	84.8	ENSMODP00000032889	1	94.3
ENSP00000365295	11	ENSPPYP00000004258	11	97.4	ENSPPYP00000004665	11	98.7
ENSP00000314023	11	ENSGALP00000019390	24	73.2	ENSGALP00000027706	1	81.3
ENSP00000227378	11	ENSRNOP00000042159	8	99.8	ENSRNOP00000048107	2	100
ENSP00000334992	11	ENSRNOP00000056844	8	69.4	ENSRNOP00000050745	8	79.9
ENSP00000237841	12	ENSPPYP00000004744	12	92.3	ENSPPYP00000016866	4	95.5
ENSP00000380148	12	ENSTNIP00000008481	8	75.2	ENSTNIP00000013704	7	79.6
ENSP00000380068	12	ENSRNOP00000025351	4	71.8	ENSRNOP00000043952	4	96.9
ENSP00000266546	12	ENSCAFP00000020767	27	36.9	ENSCAFP00000029221	5	52.1

ENSP00000339250	12	ENSPTRP00000007885	12	99.4	ENSPTRP00000056083	14	100
ENSP00000162391	12	ENSMMUP00000020004	11	98.1	ENSMMUP00000040960	Un	100
ENSP00000352233	12	ENSPTRP00000007907	12	92.8	ENSPTRP00000057421	11	96
ENSP00000350136	12	ENSMMUP00000040270	11	93.1	ENSMMUP00000036287	17	95.8
ENSP00000279544	12	ENSRNOP00000029863	4	22.3	ENSRNOP00000039380	4	30.6
ENSP00000228862	12	ENSPPYP00000004918	12	99.2	ENSPPYP00000005895	12	100
ENSP00000373730	12	ENSPTRP00000008065	12	95.2	ENSPTRP00000030444	1	96.9
ENSP00000256078	12	ENSECAP00000017062	6	91.9	ENSECAP00000015048	9	95.8
ENSP00000256078	12	ENSMODP00000021787	8	92	ENSMODP00000020698	Un	94.7
ENSP00000256682	12	ENSBTAP00000022114	5	86.2	ENSBTAP00000010159	7	96.1
ENSP00000293590	12	ENSBTAP00000022075	5	97.4	ENSBTAP00000016517	12	100
ENSP00000329995	12	ENSBTAP00000032096	5	20	ENSBTAP00000025675	5	36.5
ENSP00000352438	12	ENSRNOP00000052173	7	84.5	ENSRNOP00000037685	8	96.3
ENSP00000243082	12	ENSMODP00000011952	4	52.8	ENSMODP00000032327	8	56.9
ENSP00000307321	12	ENSTNIP00000020305	9	36.2	ENSTNIP00000004144	8	79.7
ENSP00000307321	12	ENSORLP00000009924	7	35.8	ENSORLP00000001666	15	50.4
ENSP00000368990	12	ENSECAP00000002639	6	84	ENSECAP00000004159	6	86.2
ENSP00000337632	12	ENSMMUP00000008279	11	90.4	ENSMMUP00000038767	1	92.9
ENSP00000262030	12	ENSMMUP00000001377	11	90.7	ENSMMUP00000035315	3	98.3
ENSP00000228682	12	ENSTNIP00000009858	11	45.3	ENSTNIP00000013153	3	55.7
ENSP00000266643	12	ENSORLP00000019029	5	60.1	ENSORLP00000020760	7	73.2
ENSP00000338352	12	ENSORLP00000014147	6	38.8	ENSORLP00000016965	23	77.2
ENSP00000316176	12	ENSORLP00000016944	23	85.5	ENSORLP00000014142	6	93.4
ENSP00000333275	12	ENSDARP00000066947	4	60.8	ENSDARP00000015215	8	64.4
ENSP00000008527	12	ENSORLP00000020514	6	81	ENSORLP00000020542	23	85.7
ENSP00000308083	12	ENSTNIP00000018978	12	63.5	ENSTNIP00000011930	4	65.4
ENSP00000308083	12	ENSORLP00000009503	9	61.7	ENSORLP00000010378	12	65.4
ENSP00000347464	12	ENSORLP00000009168	9	76.2	ENSORLP00000010142	12	80.3
ENSP00000228825	12	ENSMODP00000014558	3	97.8	ENSMODP00000037579	2	98.9
ENSP00000228841	12	ENSRNOP00000054295	12	66	ENSRNOP00000048768	12	78.3
ENSP00000257566	12	ENSGALP00000013415	15	89.3	ENSGALP00000038418	19	94.3
ENSP00000305059	12	ENSPTRP00000023010	12	97.6	ENSPTRP00000014400	16	99.2
ENSP00000376328	12	ENSTNIP00000018679	12	51.9	ENSTNIP00000011927	4	58.2
ENSP00000319474	12	ENSDARP00000075940	5	48.2	ENSDARP00000005989	15	51.1
ENSP00000350686	12	ENSDARP00000084954	5	51.1	ENSDARP00000062926	10	58.7
ENSP00000204726	12	ENSRNOP00000053494	12	81.8	ENSRNOP00000000865	4	87.7
ENSP00000382982	13	ENSPTRP00000021340	13	98.9	ENSPTRP00000021312	2b	100
ENSP00000241125	13	ENSMUSP00000059587	14	74.3	ENSMUSP00000102678	3	77.8
ENSP00000370877	13	ENSDARP00000085962	24	67.5	ENSDARP00000047935	21	79.3
ENSP00000370569	13	ENSMUSP00000106213	5	99.4	ENSMUSP00000111808	5	100
ENSP00000370414	13	ENSPPYP00000005968	13	86.3	ENSPPYP00000012492	20	94.1
ENSP00000345347	13	ENSRNOP00000049627	12	94.4	ENSRNOP00000051063	1	99.4
ENSP00000369191	13	ENSECAP00000008487	17	76.4	ENSECAP00000002913	X	92.9
ENSP00000347314	13	ENSTNIP00000012814	7	42.3	ENSTNIP00000019362	16	53.6
ENSP00000368477	13	ENSMMUP00000036359	17	58.7	ENSMMUP00000023277	2	89.2

ENSP00000310260	13	ENSORLP00000012719	21	65.2	ENSORLP00000012542	10	67.2
ENSP00000366819	13	ENSRNOP00000012842	15	93	ENSRNOP00000008390	3	97.8
ENSP00000366308	13	ENSTNIP00000001514	3	45.6	ENSTNIP00000016045	20	52.7
ENSP00000365895	13	ENSGALP00000025389	1	51	ENSGALP00000009781	4	73.6
ENSP00000383087	13	ENSCAFP00000008642	22	99.5	ENSCAFP00000027109	X	100
ENSP00000364552	13	ENSORLP00000005913	3	77.6	ENSORLP00000015843	21	83.8
ENSP00000308208	14	ENSORLP00000009176	18	65.3	ENSORLP00000015212	17	67.5
ENSP00000315112	14	ENSORLP00000016757	20	45.5	ENSORLP00000005328	17	47.9
ENSP00000322508	14	ENSMMUP00000004176	7	64	ENSMMUP00000033352	4	86
ENSP00000298283	14	ENSECAP00000003445	1	89.3	ENSECAP00000014927	X	95.3
ENSP00000328596	14	ENSBTAP00000017329	10	76	ENSBTAP00000038493	11	81.3
ENSP00000216513	14	ENSORLP00000019732	22	47.6	ENSORLP00000008483	13	62.2
ENSP00000261973	14	ENSRNOP00000003834	6	98.6	ENSRNOP00000003832	10	99.7
ENSP00000345395	14	ENSCAFP00000024742	8	77.1	ENSCAFP00000024739	Un	79.6
ENSP00000331474	14	ENSMODP00000032577	1	64	ENSMODP00000007807	1	70.2
ENSP00000216487	14	ENSMMUP00000018350	7	93	ENSMMUP00000039618	15	96.6
ENSP00000337133	14	ENSECAP00000021125	24	30.5	ENSECAP00000009655	X	52.3
ENSP00000384720	14	ENSORLP00000019561	22	66.2	ENSORLP00000011908	24	70.2
ENSP00000333905	14	ENSTNIP00000013904	14	84.5	ENSTNIP00000020422	10	86.2
ENSP00000251181	14	ENSTNIP00000020602	10	39.3	ENSTNIP00000007456	14	45.5
ENSP00000251181	14	ENSORLP00000021327	22	37.9	ENSORLP00000022661	24	41.8
ENSP00000300177	15	ENSDARP00000043381	20	60.2	ENSDARP00000094646	17	71.3
ENSP00000325166	15	ENSMODP00000001744	1	23.2	ENSMODP00000034985	2	40.9
ENSP00000331108	15	ENSTNIP00000015179	14	60.2	ENSTNIP00000020585	10	70.2
ENSP00000290378	15	ENSTNIP00000020592	10	98.7	ENSTNIP00000015185	14	99.5
ENSP00000382380	15	ENSTNIP00000006557	5	53.6	ENSTNIP00000013303	13	56.9
ENSP00000267812	15	ENSMODP00000033143	1	88.1	ENSMODP00000032704	8	89.7
ENSP00000261868	15	ENSMODP00000022062	1	94.4	ENSMODP00000030508	4	96.9
ENSP00000260433	15	ENSTNIP00000012393	5	50	ENSTNIP00000016690	13	53.8
ENSP00000261837	15	ENSORLP00000016014	3	79.2	ENSORLP00000006943	6	92.4
ENSP00000313299	15	ENSTNIP00000012189	5	42.8	ENSTNIP00000016706	13	51.7
ENSP00000288207	15	ENSRNOP00000017117	8	90.2	ENSRNOP00000058029	1	93.1
ENSP00000331019	15	ENSCAFP00000035017	30	92.9	ENSCAFP00000031088	7	96.4
ENSP00000332973	15	ENSTNIP00000011268	13	88.5	ENSTNIP00000012419	5	95.3
ENSP00000332973	15	ENSORLP00000008548	6	88.9	ENSORLP00000002748	3	95.5
ENSP00000332973	15	ENSDARP00000043454	18	93.8	ENSDARP00000045373	7	97.2
ENSP00000369374	15	ENSDARP00000090876	7	59.4	ENSDARP00000092063	10	77.7
ENSP00000327290	15	ENSDARP00000024989	25	55.1	ENSDARP00000052388	7	59.4
ENSP00000261917	15	ENSGALP00000002705	10	77.6	ENSGALP00000038157	Z	84
ENSP00000290438	15	ENSMMUP00000033437	7	55.6	ENSMMUP00000033372	7	83.3
ENSP00000300576	15	ENSPTRP00000041163	15	92.6	ENSPTRP00000051378	15	96.7
ENSP00000300576	15	ENSMMUP00000030995	7	41.6	ENSMMUP00000033372	7	84.9
ENSP00000360132	15	ENSRNOP00000007261	8	92.7	ENSRNOP00000057448	6	98.6
ENSP00000290759	15	ENSTNIP00000011216	13	84.1	ENSTNIP00000014302	5	88.8
ENSP00000327846	15	ENSMODP00000038560	1	8.3	ENSMODP00000001969	1	15

ENSP00000327846	15	ENSTNIP00000011240	13	13.3	ENSTNIP00000006021	5	25
ENSP00000329918	15	ENSGALP00000033501	10	58.6	ENSGALP00000001658	28	64
ENSP00000320092	15	ENSMODP00000035386	1	58.6	ENSMODP00000000267	3	71.6
ENSP00000352606	15	ENSDARP00000008101	5	49.2	ENSDARP00000067322	25	58.5
ENSP00000300233	15	ENSRNOP00000031316	1	75.2	ENSRNOP00000035266	1	79.6
ENSP00000234798	16	ENSBTAP00000009636	25	46.7	ENSBTAP00000027425	25	52.7
ENSP00000293937	16	ENSGALP00000008679	14	55.3	ENSGALP00000002089	18	73.9
ENSP00000293937	16	ENSTNIP00000015880	2	47.1	ENSTNIP00000021170	3	69.4
ENSP00000341885	16	ENSMMUP00000032726	20	66.9	ENSMMUP00000035806	9	100
ENSP00000219596	16	ENSBTAP00000025458	25	21.2	ENSBTAP00000010051	23	34.1
ENSP00000219596	16	ENSECAP00000005035	13	19.6	ENSECAP00000014005	14	33.9
ENSP00000294016	16	ENSTNIP00000023065	3	49.5	ENSTNIP00000017105	18	63.5
ENSP00000262366	16	ENSORLP00000010384	8	55.1	ENSORLP00000012982	1	71.8
ENSP00000305465	16	ENSECAP00000008763	13	75.4	ENSECAP00000017191	13	79.8
ENSP00000343488	16	ENSRNOP00000035801	1	84.8	ENSRNOP00000025584	1	87.8
ENSP00000219638	16	ENSCAFP00000026061	6	83.9	ENSCAFP00000022992	8	90.7
ENSP00000330606	16	ENSCAFP00000030413	6	64.6	ENSCAFP00000032995	6	73.8
ENSP00000322524	16	ENSRNOP00000024074	1	75.1	ENSRNOP00000058597	16	79.7
ENSP00000314030	16	ENSMMUP00000003002	20	95.4	ENSMMUP00000037614	Un	99.8
ENSP00000350054	16	ENSORLP00000017537	3	66.2	ENSORLP00000006541	16	68.6
ENSP00000381574	16	ENSRNOP00000018888	19	92.9	ENSRNOP00000058543	5	94.5
ENSP00000331608	16	ENSDARP00000069381	7	47.4	ENSDARP00000076806	16	50.4
ENSP00000342981	16	ENSMMUP00000015713	20	66.7	ENSMMUP00000035321	Un	94.9
ENSP00000353646	16	ENSMODP00000028258	1	54.5	ENSMODP00000000712	3	64.2
ENSP00000326272	16	ENSMODP00000005575	1	60.2	ENSMODP00000036576	6	69.1
ENSP00000376902	16	ENSPPYP00000008613	16	46	ENSPPYP00000008726	16	99.5
ENSP00000268695	16	ENSMMUP00000016319	20	80.8	ENSMMUP00000040661	Un	94.7
ENSP00000367609	16	ENSMMUP00000016327	20	93.9	ENSMMUP00000032970	Un	97.2
ENSP00000316809	17	ENSORLP00000012125	13	70.1	ENSORLP00000006362	14	80.7
ENSP00000347721	17	ENSTNIP00000011407	7	46.2	ENSTNIP00000009266	3	50.2
ENSP00000269260	17	ENSMODP00000007010	2	86.8	ENSMODP00000029739	3	91.2
ENSP00000007699	17	ENSTNIP00000013037	20	44.9	ENSTNIP00000002231	9	48.5
ENSP00000293829	17	ENSCAFP00000024001	5	35.5	ENSCAFP00000008879	22	72.7
ENSP00000254846	17	ENSORLP00000004718	18	69.9	ENSORLP00000012866	14	81.4
ENSP00000374011	17	ENSPTRP00000056798	17	79.8	ENSPTRP00000050919	17	95.2
ENSP00000225576	17	ENSMMUP00000035179	16	82.3	ENSMMUP00000001675	Un	87.7
ENSP00000368818	17	ENSTNIP00000021179	3	58.9	ENSTNIP00000015874	2	62
ENSP00000225388	17	ENSBTAP00000044207	19	92.4	ENSBTAP00000019593	19	93.6
ENSP00000254457	17	ENSDARP00000025458	5	77.4	ENSDARP00000021069	15	88.4
ENSP00000316426	17	ENSTNIP00000010969	6	37.4	ENSTNIP00000017157	18	42.8
ENSP00000316426	17	ENSORLP00000009074	20	36.9	ENSORLP00000003371	1	50.9
ENSP00000354033	17	ENSTNIP00000017156	18	61.9	ENSTNIP00000010967	6	66.1
ENSP00000225426	17	ENSRNOP00000017377	10	96.1	ENSRNOP00000055036	X	98.4
ENSP00000347823	17	ENSMODP00000016997	2	55.8	ENSMODP00000017996	2	63.6
ENSP00000264661	17	ENSTNIP00000014661	3	70	ENSTNIP00000007767	2	85.2

ENSP00000264661	17	ENSORLP00000004860	8	63.3	ENSORLP00000005975	11	69.8
ENSP00000253789	17	ENSBTAP00000040460	19	86.3	ENSBTAP00000027851	26	91.2
ENSP00000374035	17	ENSORLP00000003245	8	71.8	ENSORLP00000006898	19	80.6
ENSP00000007414	17	ENSTNIP00000013000	2	45.9	ENSTNIP00000002143	2	66.2
ENSP00000007414	17	ENSORLP00000015321	19	44.4	ENSORLP00000022010	21	54.2
ENSP00000007414	17	ENSDARP00000013230	12	46.3	ENSDARP00000087844	9	55
ENSP00000377060	17	ENSMMUP00000026136	16	85.2	ENSMMUP00000021644	14	98.4
ENSP00000377033	17	ENSMODP00000015401	2	85.3	ENSMODP00000000675	1	93
ENSP00000354686	17	ENSORLP00000003893	8	67	ENSORLP00000011783	20	69.5
ENSP00000285273	17	ENSCAFP00000025529	9	46.8	ENSCAFP00000005855	1	53.3
ENSP00000268919	17	ENSGALP00000004742	18	44.6	ENSGALP00000023720	Z	57.4
ENSP00000240343	17	ENSMMUP00000029586	16	78.4	ENSMMUP00000001597	Un	93.3
ENSP00000308344	17	ENSMMUP00000006858	16	72.1	ENSMMUP00000023771	Un	99.2
ENSP00000225742	17	ENSCAFP00000018718	9	91.9	ENSCAFP00000000434	1	97.3
ENSP00000205948	17	ENSMMUP00000019820	16	92.9	ENSMMUP00000017501	1	95.4
ENSP00000343900	17	ENSGALP00000007054	18	44.9	ENSGALP00000007498	14	64.8
ENSP00000308312	17	ENSBTAP00000022242	19	86.8	ENSBTAP00000045719	20	90.1
ENSP00000306761	17	ENSRNOP00000004718	10	95.3	ENSRNOP00000044615	19	98.9
ENSP00000310590	17	ENSTNIP00000012663	3	53.2	ENSTNIP00000000995	2	61.6
ENSP00000262768	17	ENSDARP00000055088	3	53.3	ENSDARP00000066228	12	75.1
ENSP00000363875	17	ENSPTRP00000016624	17	63.2	ENSPTRP00000008072	12	81.7
ENSP00000386002	17	ENSTNIP00000012655	3	75.2	ENSTNIP00000017060	18	84.5
ENSP00000304283	17	ENSCAFP00000008868	9	75.5	ENSCAFP00000002174	10	94
ENSP00000304283	17	ENSDARP00000044689	12	96.4	ENSDARP00000059520	3	97.9
ENSP00000331500	17	ENSBTAP00000020343	19	65.2	ENSBTAP00000011370	3	70.1
ENSP00000261596	18	ENSORLP00000014179	20	62.2	ENSORLP00000022357	17	64.4
ENSP00000348821	18	ENSORLP00000022349	17	55.9	ENSORLP00000014276	20	59.8
ENSP00000352927	18	ENSTNIP00000017902	15	52.3	ENSTNIP00000022297	8	56.6
ENSP00000352927	18	ENSORLP00000004892	17	52	ENSORLP00000007176	16	54.4
ENSP00000262126	18	ENSORLP00000005005	17	65.4	ENSORLP00000007166	3	76.1
ENSP00000019317	18	ENSRNOP00000033120	9	92	ENSRNOP00000058746	7	93.7
ENSP00000304908	18	ENSMUSP00000054909	17	48.9	ENSMUSP00000030051	4	61
ENSP00000304908	18	ENSCAFP00000027629	7	49.2	ENSCAFP00000004307	11	58.3
ENSP00000304908	18	ENSBTAP00000022959	24	52.7	ENSBTAP00000041860	8	62.9
ENSP00000269214	18	ENSRNOP00000019042	18	88.3	ENSRNOP00000049071	13	93.9
ENSP00000372612	18	ENSMMUP00000008854	18	91.9	ENSMMUP00000011370	15	96
ENSP00000372528	18	ENSECAP00000005380	8	75.7	ENSECAP00000001115	8	78.9
ENSP00000338217	18	ENSGALP00000004487	Z	83.4	ENSGALP00000011430	20	92.6
ENSP00000299502	18	ENSBTAP00000031506	24	77.1	ENSBTAP00000031243	24	79.3
ENSP00000262150	18	ENSORLP00000015798	20	43.6	ENSORLP00000001707	15	54.3
ENSP00000353377	18	ENSGALP00000030322	2	44	ENSGALP00000022037	11	52.4
ENSP00000348273	18	ENSMMUP00000041140	18	97.3	ENSMMUP00000014872	Un	100
ENSP00000299466	18	ENSDARP00000074744	19	56.3	ENSDARP00000076069	16	63.2
ENSP00000315654	18	ENSDARP00000052196	19	63.5	ENSDARP00000082184	23	72.5
ENSP00000251287	19	ENSCAFP00000029177	20	88.2	ENSCAFP00000026255	30	93.5

ENSP00000251287	19	ENSGALP00000002046	28	76.3	ENSGALP00000038157	Z	84.3
ENSP00000347886	19	ENSTNIP00000018387	15	63.9	ENSTNIP00000013540	1	82.5
ENSP00000349575	19	ENSMMUP00000031998	19	64.6	ENSMMUP00000014394	15	80.2
ENSP00000252622	19	ENSMMUP00000000610	19	47.3	ENSMMUP00000032578	Un	95.9
ENSP00000350325	19	ENSMMUP00000031967	19	51	ENSMMUP00000026775	7	56.6
ENSP00000307940	19	ENSMODP00000000901	3	97.8	ENSMODP00000037758	1	98.6
ENSP00000317912	19	ENSDARP00000091289	8	34	ENSDARP00000069435	2	44.9
ENSP00000301293	19	ENSMODP00000032639	3	47.7	ENSMODP00000038010	6	58.2
ENSP00000264071	19	ENSMODP00000018721	3	93.9	ENSMODP00000021471	1	98.6
ENSP00000352814	19	ENSGALP00000000878	28	40.4	ENSGALP00000016496	Z	44.4
ENSP00000302867	19	ENSMUSP00000072248	9	61.4	ENSMUSP00000083680	9	77.6
ENSP00000310488	19	ENSMMUP00000031869	19	58.9	ENSMMUP00000027591	Un	92.2
ENSP00000250244	19	ENSMMUP00000031846	19	95.7	ENSMMUP00000033945	Un	99.3
ENSP00000242784	19	ENSRNOP00000005270	19	93	ENSRNOP00000053710	14	96.1
ENSP00000253673	19	ENSECAP00000009252	7	58.1	ENSECAP00000019713	3	73.9
ENSP00000300935	19	ENSORLP00000022571	17	88.8	ENSORLP00000021325	4	91.3
ENSP00000252773	19	ENSMMUP00000020302	19	68	ENSMMUP00000021422	15	78.3
ENSP00000323332	19	ENSDARP00000070472	20	65	ENSDARP00000088655	2	82.7
ENSP00000222271	19	ENSGALP00000005183	28	74.9	ENSGALP00000037843	Z	78.4
ENSP00000162023	19	ENSTNIP00000016179	1	35.6	ENSTNIP00000022430	13	59
ENSP00000252575	19	ENSORLP00000001026	4	41.4	ENSORLP00000000500	12	54.5
ENSP00000251203	19	ENSBTAP00000013152	7	75.2	ENSBTAP00000019181	23	84.1
ENSP00000329991	19	ENSECAP00000003580	10	70.7	ENSECAP00000002978	22	75.9
ENSP00000384995	19	ENSMODP00000026094	4	21.5	ENSMODP00000038220	Un	41.8
ENSP00000333595	19	ENSRNOP00000026088	1	44.5	ENSRNOP00000039434	1	48.9
ENSP00000300853	19	ENSCAFP00000006636	1	88.2	ENSCAFP00000031162	17	93.9
ENSP00000384318	19	ENSPTRP00000019152	19	20.9	ENSPTRP00000010871	14	44.9
ENSP00000342374	19	ENSCAFP00000006557	1	71.7	ENSCAFP00000000897	13	91.5
ENSP00000324820	19	ENSORLP00000008705	13	67.7	ENSORLP00000011170	14	69.5
ENSP00000236877	19	ENSECAP00000006676	10	64.1	ENSECAP00000005065	15	74
ENSP00000313309	19	ENSMMUP00000015244	19	97.8	ENSMMUP00000039295	Un	98.7
ENSP00000293441	19	ENSECAP00000015633	10	68.7	ENSECAP00000020723	12	73.2
ENSP00000270464	19	ENSMUSP00000019416	7	49	ENSMUSP00000101122	10	52.6
ENSP00000320390	19	ENSMODP00000003499	4	37.7	ENSMODP00000008411	Un	44.9
ENSP00000346265	19	ENSORLP00000008623	19	55.2	ENSORLP00000020459	23	58.4
ENSP00000307863	19	ENSMMUP00000006397	19	92.8	ENSMMUP00000035632	Un	99.5
ENSP00000379451	19	ENSECAP00000012406	10	56.2	ENSECAP00000000336	10	61.9
ENSP00000383003	20	ENSCAFP00000010138	24	90.7	ENSCAFP00000032061	12	97.2
ENSP00000347076	20	ENSMODP00000006630	1	84.6	ENSMODP00000005948	8	87
ENSP00000278886	20	ENSMODP00000008056	1	55	ENSMODP00000036523	4	78.3
ENSP00000365145	20	ENSMODP00000024306	1	58.6	ENSMODP00000036576	6	64.9
ENSP00000362354	20	ENSGALP00000005986	20	55.8	ENSGALP00000036088	2	72.6
ENSP00000217043	20	ENSDARP00000039704	23	61.7	ENSDARP00000081731	2	65.7
ENSP00000255132	20	ENSMMUP00000004537	10	90.2	ENSMMUP00000004538	Un	96
ENSP00000361133	20	ENSGALP00000007168	20	81.4	ENSGALP00000025136	2	85

ENSP00000252998	20	ENSMODP00000020925	1	50.9	ENSMODP00000004615	3	55.3
ENSP00000350864	20	ENSECAP00000018782	22	67.6	ENSECAP00000015861	9	72.1
ENSP00000333194	20	ENSGALP00000033269	20	38.1	ENSGALP00000035975	2	82.4
ENSP00000358783	20	ENSMUSP00000071513	2	36.7	ENSMUSP00000046233	1	61.8
ENSP00000358783	20	ENSRNOP00000051902	3	39.8	ENSRNOP00000010032	5	61.8
ENSP00000353959	21	ENSMUSP00000023670	16	70	ENSMUSP00000041453	4	73.4
ENSP00000353959	21	ENSRNOP00000033206	11	70.1	ENSRNOP00000024464	5	73.4
ENSP00000383333	21	ENSTNIP00000016946	2	45.1	ENSTNIP00000019894	1	57.4
ENSP00000291565	21	ENSPPYP00000012808	21	70.5	ENSPPYP00000022383	Un	94
ENSP00000253413	22	ENSTNIP00000011645	13	72.1	ENSTNIP00000014358	19	79.2
ENSP00000329393	22	ENSRNOP00000026477	14	81.8	ENSRNOP00000037079	4	85.7
ENSP00000216044	22	ENSPPYP00000013192	22	98.7	ENSPPYP00000013394	22	99.3
ENSP00000327545	22	ENSTNIP00000022026	18	45.4	ENSTNIP00000015867	2	53.4
ENSP00000216237	22	ENSORLP00000003118	19	50.2	ENSORLP00000018417	19	60.6
ENSP00000263243	22	ENSORLP00000017714	8	42.9	ENSORLP00000006128	8	48
ENSP00000216264	22	ENSTNIP00000013389	19	55.3	ENSTNIP00000011277	13	58.8
ENSP00000216268	22	ENSMUSP00000035437	15	82.3	ENSMUSP00000111895	12	85.1
ENSP00000252783	22	ENSPTRP00000057422	22	31.7	ENSPTRP00000053250	8	43.4

Table S4. 250 putative true events

Human reference protein	Chr	Vertebrate synteny ortholog	Chr	%ID	Vertebrate similarity ortholog	Chr	%ID
ENSP00000306330	7	ENSTNIP00000001215	7	93.9	ENSTNIP00000019330	16	96.8
ENSP00000379278	8	ENSORLP00000003299	16	79.5	ENSORLP00000012885	7	87.3
ENSP00000383493	c6	ENSPTRP00000055488	6	67.5	ENSPTRP00000030539	6	94.3
ENSP00000333905	14	ENSTNIP00000013904	14	84.5	ENSTNIP00000020422	10	86.2
ENSP00000372713	c6	ENSPPYP00000019310	6	53.8	ENSPPYP00000018450	6	97.6
ENSP00000263817	2	ENSORLP00000021369	21	66.6	ENSORLP00000004294	2	70.1
ENSP00000331108	15	ENSTNIP00000015179	14	60.2	ENSTNIP00000020585	10	70.2
ENSP00000290378	15	ENSTNIP00000020592	10	98.7	ENSTNIP00000015185	14	99.5
ENSP00000360165	X	ENSMUSP00000062570	X	70.9	ENSMUSP00000050377	4	74.2
ENSP00000262126	18	ENSORLP00000005005	17	65.4	ENSORLP00000007166	3	76.1
ENSP00000379890	2	ENSTNIP00000017321	16	59.7	ENSTNIP00000009139	1	72
ENSP00000364126	X	ENSRNOP00000000179	X	65.4	ENSRNOP00000037807	14	73.6
ENSP00000256682	12	ENSBTAP00000022114	5	86.2	ENSBTAP00000010159	7	96.1
ENSP00000228825	12	ENSMODP00000014558	3	97.8	ENSMODP00000037579	2	98.9
ENSP00000370414	13	ENSPPYP00000005968	13	86.3	ENSPPYP00000012492	20	94.1
ENSP00000377033	17	ENSMODP00000015401	2	85.3	ENSMODP00000000675	1	93
ENSP00000374035	17	ENSORLP00000003245	8	71.8	ENSORLP00000006898	19	80.6
ENSP00000262030	12	ENSMMUP00000001377	11	90.7	ENSMMUP00000035315	3	98.3
ENSP00000292475	7	ENSMMUP00000025227	3	95.7	ENSMMUP00000038266	12	97.9
ENSP00000321507	8	ENSDARP00000069192	16	53.3	ENSDARP00000052078	19	55.8
ENSP00000263610	9	ENSDARP00000089097	5	80.1	ENSDARP00000016114	21	82.5
ENSP00000005260	7	ENSORLP00000011904	8	54.1	ENSORLP00000004171	19	56.4
ENSP00000324960	11	ENSMODP00000009288	8	61.1	ENSMODP00000026669	Un	68.5
ENSP00000315299	10	ENSECAP00000004472	29	79.9	ENSECAP00000006446	15	84.6
ENSP00000384974	2	ENSDARP00000022350	13	99.4	ENSDARP00000091642	1	100
ENSP00000363939	9	ENSMUSP00000029991	4	80.6	ENSMUSP00000099944	11	84.5
ENSP00000301920	2	ENSTNIP00000013266	3	50.7	ENSTNIP00000009491	2	54.4
ENSP00000377060	17	ENSMMUP00000026136	16	85.2	ENSMMUP00000021644	14	98.4
ENSP00000385991	2	ENSGALP00000012796	3	17.6	ENSGALP00000018146	5	78.1
ENSP00000377547	5	ENSCAFP00000025446	4	85.7	ENSCAFP00000013853	32	88.9
ENSP00000374011	17	ENSPTRP00000056798	17	79.8	ENSPTRP00000050919	17	95.2
ENSP00000216264	22	ENSTNIP00000013389	19	55.3	ENSTNIP00000011277	13	58.8
ENSP00000326624	6	ENSBTAP00000022407	9	8.2	ENSBTAP00000008495	1	15.9
ENSP00000301896	11	ENSMMUP00000037490	14	53.9	ENSMMUP00000041118	Un	97.1
ENSP00000364219	1	ENSDARP00000068888	6	66.2	ENSDARP00000035352	2	76.9
ENSP00000311083	1	ENSMMUP00000008659	1	92.5	ENSMMUP00000036256	17	97.5
ENSP00000353959	21	ENSMUSP00000023670	16	70	ENSMUSP00000041453	4	73.4
ENSP00000353959	21	ENSRNOP00000033206	11	70.1	ENSRNOP00000024464	5	73.4
ENSP00000361616	10	ENSTNIP00000011470	17	43.3	ENSTNIP00000016145	2	49.3
ENSP00000309762	3	ENSPTRP00000026513	3	98.2	ENSPTRP00000035127	8	100
ENSP00000329419	3	ENSORLP00000019501	13	74.4	ENSORLP00000017646	17	90.3
ENSP00000260433	15	ENSTNIP00000012393	5	50	ENSTNIP00000016690	13	53.8
ENSP00000306761	17	ENSRNOP00000004718	10	95.3	ENSRNOP00000044615	19	98.9
ENSP00000182096	3	ENSMODP00000021223	7	66.9	ENSMODP00000003367	4	75.8
ENSP00000305964	4	ENSMUSP00000060890	5	58.8	ENSMUSP00000033582	X	70
ENSP00000305964	4	ENSRNOP00000056258	14	62.3	ENSRNOP00000054370	X	70

ENSP00000358358	6	ENSORLP00000022430	24	45.7	ENSORLP00000013911	22	70
ENSP00000381574	16	ENSRNOP00000018888	19	92.9	ENSRNOP00000058543	5	94.5
ENSP00000334330	8	ENSMUSP00000076067	8	58.1	ENSMUSP00000060899	8	66.7
ENSP00000364552	13	ENSORLP00000005913	3	77.6	ENSORLP00000015843	21	83.8
ENSP00000382840	X	ENSDARP00000002260	9	81.5	ENSDARP00000010461	6	83.5
ENSP00000369127	9	ENSECAP00000008597	23	99.2	ENSECAP00000002642	13	100
ENSP00000262177	7	ENSMODP00000006478	8	78.8	ENSMODP00000029650	2	83.3
ENSP00000353701	11	ENSRNOP00000026968	1	84.6	ENSRNOP00000007288	3	93.4
ENSP00000341680	6	ENSDARP00000094783	19	61.5	ENSDARP00000071887	16	65.3
ENSP00000343244	X	ENSBTAP00000002365	X	65.1	ENSBTAP00000023205	17	69.7
ENSP00000240343	17	ENSMMUP00000029586	16	78.4	ENSMMUP00000001597	Un	93.3
ENSP00000307940	19	ENSMODP00000000901	3	97.8	ENSMODP00000037758	1	98.6
ENSP00000261868	15	ENSMODP00000022062	1	94.4	ENSMODP00000030508	4	96.9
ENSP00000304736	4	ENSMODP00000038335	5	70.7	ENSMODP00000016038	2	95.8
ENSP00000269214	18	ENSRNOP00000019042	18	88.3	ENSRNOP00000049071	13	93.9
ENSP00000309982	4	ENSBTAP00000006095	17	32.7	ENSBTAP00000049751	22	43.9
ENSP00000253490	5	ENSPTRP00000029981	5	93.8	ENSPTRP00000040688	5	95.5
ENSP00000329995	12	ENSBTAP00000032096	5	20	ENSBTAP00000025675	5	36.5
ENSP00000295569	3	ENSTNIP00000014063	11	64.1	ENSTNIP00000018093	9	79.2
ENSP00000357086	1	ENSMUSP00000091861	1	24	ENSMUSP00000036380	1	37.3
ENSP00000378005	5	ENSTNIP00000021373	7	50.4	ENSTNIP00000011799	1	56.5
ENSP00000314299	1	ENSPTRP00000003010	1	86.3	ENSPTRP00000044519	1	96.1
ENSP00000383003	20	ENSCAFP00000010138	24	90.7	ENSCAFP00000032061	12	97.2
ENSP00000384196	6	ENSECAP00000002730	31	95.2	ENSECAP00000003082	9	97.4
ENSP00000347041	1	ENSORLP00000019181	23	45.8	ENSORLP00000019666	5	50.6
ENSP00000326272	16	ENSMODP00000005575	1	60.2	ENSMODP00000036576	6	69.1
ENSP00000367284	X	ENSMMUP00000004669	X	88.4	ENSMMUP00000004448	6	97.4
ENSP00000383007	4	ENSORLP00000000360	10	45.2	ENSORLP00000002647	7	52.6
ENSP00000232564	3	ENSRNOP00000014871	2	77.3	ENSRNOP00000022649	5	90.9
ENSP00000261837	15	ENSORLP00000016014	3	79.2	ENSORLP00000006943	6	92.4
ENSP00000260950	2	ENSORLP00000018869	21	61.5	ENSORLP00000018605	5	64.8
ENSP00000379497	8	ENSORLP00000011160	16	53.4	ENSORLP00000006814	3	59.6
ENSP00000364107	X	ENSMMUP00000023726	X	81.9	ENSMMUP00000003292	1	86.2
ENSP00000351706	4	ENSRNOP00000045775	14	83	ENSRNOP00000047647	X	88.4
ENSP00000290438	15	ENSMMUP00000033437	7	55.6	ENSMMUP00000033372	7	83.3
ENSP00000300576	15	ENSMMUP00000030995	7	41.6	ENSMMUP00000033372	7	84.9
ENSP00000300576	15	ENSPTRP00000041163	15	92.6	ENSPTRP00000051378	15	96.7
ENSP00000359634	10	ENSORLP00000009559	15	73.2	ENSORLP00000014372	1	81.3
ENSP00000234160	2	ENSTNIP00000020049	2	59.6	ENSTNIP00000010714	15	70.6
ENSP00000318650	1	ENSDARP00000072950	17	62.7	ENSDARP00000008325	12	70.5
ENSP00000324820	19	ENSORLP00000008705	13	67.7	ENSORLP00000011170	14	69.5
ENSP00000344984	11	ENSPPYP00000004050	11	16.1	ENSPPYP00000019730	7	27.4
ENSP00000307705	6	ENSRNOP00000022735	17	57.1	ENSRNOP00000024304	17	85.5
ENSP00000373730	12	ENSPTRP00000008065	12	95.2	ENSPTRP00000030444	1	96.9
ENSP00000350580	6	ENSBTAP00000036521	23	98.5	ENSBTAP00000053002	3	100
ENSP00000364398	9	ENSTNIP00000000397	12	41.6	ENSTNIP00000009737	15	44.6
ENSP00000230012	6	ENSMUSP00000057557	13	52.6	ENSMUSP00000005017	3	58.5
ENSP00000358414	1	ENSGALP00000004672	8	64.7	ENSGALP00000023936	Z	67.1
ENSP00000295206	2	ENSDARP00000023487	9	52.8	ENSDARP00000094379	1	62.6
ENSP00000345347	13	ENSRNOP00000049627	12	94.4	ENSRNOP00000051063	1	99.4

ENSP00000381654	6	ENSTNIP00000019668	17	62.9	ENSTNIP00000000883	10	65.7
ENSP00000317788	9	ENSMODP00000010448	6	99.1	ENSMODP00000023704	5	99.8
ENSP00000352606	15	ENSDARP00000008101	5	49.2	ENSDARP00000067322	25	58.5
ENSP00000293937	16	ENSGALP00000008679	14	55.3	ENSGALP00000002089	18	73.9
ENSP00000368046	4	ENSMMUP00000002781	5	81	ENSMMUP00000002782	7	89.6
ENSP00000222718	7	ENSDARP00000074915	19	30	ENSDARP00000023072	16	72.1
ENSP00000353151	7	ENSECAP00000000275	4	34.1	ENSECAP00000003063	6	52.9
ENSP00000307321	12	ENSORLP00000009924	7	35.8	ENSORLP00000001666	15	50.4
ENSP00000243082	12	ENSMODP00000011952	4	52.8	ENSMODP00000032327	8	56.9
ENSP00000340281	11	ENSTNIP00000012231	5	71.9	ENSTNIP00000016721	13	83.6
ENSP00000382707	11	ENSMMUP00000017430	14	94.7	ENSMMUP00000032893	7	97
ENSP00000369562	9	ENSPPYP00000021489	9	81.5	ENSPPYP00000022287	9	94.1
ENSP00000357881	10	ENSORLP00000011937	19	55	ENSORLP00000007006	15	71.1
ENSP00000329991	19	ENSECAP00000003580	10	70.7	ENSECAP00000002978	22	75.9
ENSP00000310260	13	ENSORLP00000012719	21	65.2	ENSORLP00000012542	10	67.2
ENSP00000343885	4	ENSORLP00000008244	1	58.9	ENSORLP00000008491	15	61.9
ENSP00000367139	5	ENSMUSP00000062976	18	23.2	ENSMUSP00000069247	18	38.3
ENSP00000290759	15	ENSTNIP00000011216	13	84.1	ENSTNIP00000014302	5	88.8
ENSP00000327290	15	ENSDARP00000024989	25	55.1	ENSDARP00000052388	7	59.4
ENSP00000264106	2	ENSORLP00000021757	21	51.6	ENSORLP00000018008	17	54.4
ENSP00000251181	14	ENSORLP00000021327	22	37.9	ENSORLP00000022661	24	41.8
ENSP00000352927	18	ENSORLP00000004892	17	52	ENSORLP00000007176	16	54.4
ENSP00000352927	18	ENSTNIP00000017902	15	52.3	ENSTNIP00000022297	8	56.6
ENSP00000372528	18	ENSECAP00000005380	8	75.7	ENSECAP00000001115	8	78.9
ENSP00000347823	17	ENSMODP00000016997	2	55.8	ENSMODP00000017996	2	63.6
ENSP00000371740	9	ENSPPYP00000021572	9	97.4	ENSPPYP00000022286	9	99.4
ENSP00000314520	1	ENSORLP00000010656	7	86.7	ENSORLP00000008061	5	90.7
ENSP00000297814	9	ENSTNIP00000008747	12	37	ENSTNIP00000012160	5	43.5
ENSP00000252783	22	ENSPTRP00000057422	22	31.7	ENSPTRP00000053250	8	43.4
ENSP00000361373	1	ENSGALP00000032535	8	23.1	ENSGALP00000036579	4	37.5
ENSP00000374788	2	ENSMMUP00000036337	13	90.2	ENSMMUP00000006018	13	95.9
ENSP00000357973	6	ENSDARP00000062977	20	54.8	ENSDARP00000023654	17	67.2
ENSP00000200639	X	ENSORLP00000001324	10	33.9	ENSORLP00000015872	21	39.4
ENSP00000361101	9	ENSMMUP00000001702	15	88.1	ENSMMUP00000023552	15	93.2
ENSP00000354616	10	ENSORLP00000010541	15	88.5	ENSORLP00000015489	1	93.9
ENSP00000306772	4	ENSDARP00000043555	1	71.2	ENSDARP00000054371	15	93.2
ENSP00000355296	10	ENSORLP00000003266	15	52.8	ENSORLP00000013126	19	59.1
ENSP00000254457	17	ENSDARP00000025458	5	77.4	ENSDARP00000021069	15	88.4
ENSP00000270464	19	ENSMUSP00000019416	7	49	ENSMUSP00000101122	10	52.6
ENSP00000320390	19	ENSMODP00000003499	4	37.7	ENSMODP00000008411	Un	44.9
ENSP00000272748	2	ENSDARP00000087622	9	54.7	ENSDARP00000011283	6	58.6
ENSP00000243314	X	ENSCAFP00000028200	X	46.3	ENSCAFP00000033026	X	54.6
ENSP00000233121	2	ENSTNIP00000022333	14	69	ENSTNIP00000022494	10	78.9
ENSP00000266643	12	ENSORLP00000019029	5	60.1	ENSORLP00000020760	7	73.2
ENSP00000348273	18	ENSMMUP00000041140	18	97.3	ENSMMUP00000014872	Un	100
ENSP00000372612	18	ENSMMUP00000008854	18	91.9	ENSMMUP00000011370	15	96
ENSP00000360367	X	ENSMODP00000012972	X	85.6	ENSMODP00000037142	5	95
ENSP00000376379	2	ENSORLP00000021635	21	41.3	ENSORLP00000004128	8	47.7
ENSP00000349785	1	ENSMMUP00000037496	1	90.5	ENSMMUP00000020886	16	93.9
ENSP00000308208	14	ENSORLP00000009176	18	65.3	ENSORLP00000015212	17	67.5

ENSP00000383333	21	ENSTNIP00000016946	2	45.1	ENSTNIP00000019894	1	57.4
ENSP00000305059	12	ENSPTRP00000023010	12	97.6	ENSPTRP00000014400	16	99.2
ENSP00000218316	X	ENSGALP00000014739	4	43.9	ENSGALP00000027791	1	47
ENSP00000218316	X	ENSMODP00000007121	X	42.5	ENSMODP00000006150	5	47.4
ENSP00000321445	8	ENSORLP00000010073	20	58.4	ENSORLP00000020645	24	64
ENSP00000308083	12	ENSORLP00000009503	9	61.7	ENSORLP00000010378	12	65.4
ENSP00000308083	12	ENSTNIP00000018978	12	63.5	ENSTNIP00000011930	4	65.4
ENSP00000348821	18	ENSORLP00000022349	17	55.9	ENSORLP00000014276	20	59.8
ENSP00000356320	6	ENSRNOP00000030143	1	20.5	ENSRNOP00000004694	13	25.3
ENSP00000361544	X	ENSMUSP00000098772	X	45.6	ENSMUSP00000056785	7	53.6
ENSP00000361544	X	ENSRNOP00000045126	X	46.3	ENSRNOP00000027278	1	53.6
ENSP00000258775	7	ENSMODP00000033236	6	28	ENSMODP00000024778	Un	68.2
ENSP00000258775	7	ENSMUSP00000049490	11	53.5	ENSMUSP00000073532	10	64
ENSP00000258775	7	ENSRNOP00000047425	14	53.8	ENSRNOP00000003550	7	64
ENSP00000357840	6	ENSMODP00000029578	2	64.6	ENSMODP00000010832	Un	67.1
ENSP00000326806	3	ENSMODP00000019849	7	88.5	ENSMODP00000029243	2	91.7
ENSP00000339283	5	ENSORLP00000003460	12	55.4	ENSORLP00000002985	4	64.6
ENSP00000358783	20	ENSMUSP00000071513	2	36.7	ENSMUSP00000046233	1	61.8
ENSP00000296930	5	ENSCAFP00000024874	4	91.8	ENSCAFP00000008796	17	97.8
ENSP00000296930	5	ENSMMUP00000020738	6	93.5	ENSMMUP00000030737	11	99.6
ENSP00000333275	12	ENSDARP00000066947	4	60.8	ENSDARP00000015215	8	64.4
ENSP00000324687	1	ENSPTRP00000050949	1	56.2	ENSPTRP00000003749	1	94.4
ENSP00000322724	11	ENSMUSP00000065147	7	76.5	ENSMUSP00000095823	7	82.7
ENSP00000341581	11	ENSMUSP00000102472	7	55.3	ENSMUSP00000102489	7	61.6
ENSP00000341581	11	ENSRNOP00000023007	1	54.3	ENSRNOP00000021468	1	61.9
ENSP00000323224	11	ENSRNOP00000050895	1	66.8	ENSRNOP00000049559	1	88.5
ENSP00000295266	4	ENSMUSP00000060774	3	74.7	ENSMUSP00000033662	X	86
ENSP00000295266	4	ENSRNOP00000021719	2	75.3	ENSRNOP00000030279	X	86
ENSP00000297913	9	ENSCAFP00000030735	9	47.4	ENSCAFP00000033821	20	54.9
ENSP00000366378	6	ENSBTAP00000051771	23	54.4	ENSBTAP00000051036	23	88.5
ENSP00000299459	11	ENSECAP00000003406	7	83	ENSECAP00000001291	7	85.1
ENSP00000355435	1	ENSMMUP00000037562	1	71.9	ENSMMUP00000037471	Un	90.4
ENSP00000373194	3	ENSMUSP00000078873	16	76.8	ENSMUSP00000073737	16	81
ENSP00000368990	12	ENSECAP00000002639	6	84	ENSECAP00000004159	6	86.2
ENSP00000357127	1	ENSCAFP00000034756	38	79.4	ENSCAFP00000035448	Un	84.1
ENSP00000302867	19	ENSMUSP00000072248	9	61.4	ENSMUSP00000083680	9	77.6
ENSP00000279783	11	ENSECAP00000004896	12	84.3	ENSECAP00000003696	12	86.6
ENSP00000323853	11	ENSECAP00000004896	12	63.5	ENSECAP00000005325	12	83.1
ENSP00000386148	7	ENSECAP00000002543	4	74.2	ENSECAP00000005535	4	80.6
ENSP00000308361	3	ENSORLP00000000420	13	34.1	ENSORLP00000007515	13	43.7
ENSP00000386002	17	ENSTNIP00000012655	3	75.2	ENSTNIP00000017060	18	84.5
ENSP00000299502	18	ENSBTAP00000031506	24	77.1	ENSBTAP00000031243	24	79.3
ENSP00000321326	5	ENSTNIP00000002202	4	41.2	ENSTNIP00000018495	12	50.2
ENSP00000352438	12	ENSRNOP00000052173	7	84.5	ENSRNOP00000037685	8	96.3
ENSP00000305465	16	ENSECAP00000008763	13	75.4	ENSECAP00000017191	13	79.8
ENSP00000356438	1	ENSDARP00000090671	2	68.5	ENSDARP00000003684	20	73.3
ENSP00000316809	17	ENSORLP00000012125	13	70.1	ENSORLP00000006362	14	80.7
ENSP00000347004	4	ENSMODP00000007179	5	56.2	ENSMODP00000017945	1	66.3
ENSP00000347883	11	ENSORLP00000001572	6	51.4	ENSORLP00000008406	3	57.7
ENSP00000353646	16	ENSMODP00000028258	1	54.5	ENSMODP00000000712	3	64.2

ENSP00000376115	2	ENSORLP00000006086	11	58.2	ENSORLP00000020658	16	65.6
ENSP00000285094	3	ENSORLP00000006086	11	66.2	ENSORLP00000020658	16	78.8
ENSP00000310117	11	ENSMMUP00000037437	14	58.2	ENSMMUP00000006532	8	97.3
ENSP00000282412	2	ENSDARP00000011295	13	76.7	ENSDARP00000011459	12	83.2
ENSP00000282412	2	ENSTNIP00000010277	17	74.1	ENSTNIP00000008010	2	78.5
ENSP00000384318	19	ENSPTRP00000019152	19	20.9	ENSPTRP00000010871	14	44.9
ENSP00000365266	1	ENSMUSP00000043718	4	35.5	ENSMUSP00000102760	4	49.1
ENSP00000365266	1	ENSRNOP00000033783	5	35.5	ENSRNOP00000041054	14	45.3
ENSP00000365343	1	ENSPTRP00000000336	1	95.6	ENSPTRP00000000337	Un	96.9
ENSP00000328915	1	ENSPTRP00000040832	1	58.9	ENSPTRP00000052839	1	97.5
ENSP00000384995	19	ENSMODP00000026094	4	21.5	ENSMODP00000038220	Un	41.8
ENSP00000300935	19	ENSORLP00000022571	17	88.8	ENSORLP00000021325	4	91.3
ENSP00000304283	17	ENSCAFP00000008868	9	75.5	ENSCAFP00000002174	10	94
ENSP00000304283	17	ENSDARP00000044689	12	96.4	ENSDARP00000059520	3	97.9
ENSP00000348786	1	ENSMODP00000001599	2	93	ENSMODP00000009084	8	95.6
ENSP00000319096	3	ENSORLP00000007391	13	91.8	ENSORLP00000019850	17	94
ENSP00000256078	12	ENSECAP00000017062	6	91.9	ENSECAP00000015048	9	95.8
ENSP00000232217	3	ENSDARP00000048837	2	67.9	ENSDARP00000093236	15	75.4
ENSP00000265538	3	ENSPPYP00000015522	3	81.8	ENSPPYP00000011947	1	98.5
ENSP00000308820	11	ENSORLP00000008603	3	40.7	ENSORLP00000001439	6	47.2
ENSP00000365926	9	ENSMMUP00000029000	15	72.7	ENSMMUP00000019468	X	92.7
ENSP00000298283	14	ENSECAP00000003445	1	89.3	ENSECAP00000014927	X	95.3
ENSP00000370569	13	ENSMUSP00000106213	5	99.4	ENSMUSP00000111808	5	100
ENSP00000265100	5	ENSBTAP00000020092	20	97.2	ENSBTAP00000016378	19	98.6
ENSP00000265100	5	ENSMODP00000031188	1	95.2	ENSMODP00000009945	2	98.6
ENSP00000264258	2	ENSMMUP00000012930	13	95	ENSMMUP00000012975	Un	99.2
ENSP00000378163	4	ENSRNOP00000009046	2	86.2	ENSRNOP00000054703	17	98.3
ENSP00000352709	2	ENSRNOP00000033369	9	83.5	ENSRNOP00000043624	5	98.9
ENSP00000361962	6	ENSMMUP00000028077	4	97.6	ENSMMUP00000012698	7	99.2
ENSP00000315112	14	ENSORLP00000016757	20	45.5	ENSORLP00000005328	17	47.9
ENSP00000347271	6	ENSRNOP00000000582	20	87	ENSRNOP00000046992	10	97
ENSP00000361435	10	ENSCAFP00000022943	4	98.5	ENSCAFP00000022283	Un	100
ENSP00000361435	10	ENSPPYP00000002679	10	99.2	ENSPPYP00000006043	13	100
ENSP00000310785	3	ENSPPYP00000015834	3	95.8	ENSPPYP00000010163	18	97.2
ENSP00000362336	10	ENSORLP00000008519	15	82.3	ENSORLP00000001135	14	89.9
ENSP00000342940	5	ENSTNIP00000016470	4	72	ENSTNIP00000018449	12	76.6
ENSP00000265362	7	ENSORLP00000011874	6	73.5	ENSORLP00000018857	23	75.8
ENSP00000327197	3	ENSRNOP00000035285	11	83.8	ENSRNOP00000056000	2	98.2
ENSP00000368477	13	ENSMMUP00000036359	17	58.7	ENSMMUP00000023277	2	89.2
ENSP00000354590	6	ENSMUSP00000095028	17	82.4	ENSMUSP00000090857	11	91.2
ENSP00000320092	15	ENSMODP00000035386	1	58.6	ENSMODP00000000267	3	71.6
ENSP00000332973	15	ENSDARP00000043454	18	93.8	ENSDARP00000045373	7	97.2
ENSP00000332973	15	ENSORLP00000008548	6	88.9	ENSORLP00000002748	3	95.5
ENSP00000332973	15	ENSTNIP00000011268	13	88.5	ENSTNIP00000012419	5	95.3
ENSP00000342374	19	ENSCAFP00000006557	1	71.7	ENSCAFP00000000897	13	91.5
ENSP00000310620	11	ENSBTAP00000051015	29	61.6	ENSBTAP00000024786	21	73.5
ENSP00000356591	1	ENSMODP00000007588	2	74	ENSMODP00000018793	1	76
ENSP00000324948	11	ENSORLP00000001551	6	60.6	ENSORLP00000008419	3	65.7
ENSP00000337133	14	ENSECAP00000021125	24	30.5	ENSECAP00000009655	X	52.3
ENSP00000365811	10	ENSMUSP00000092751	2	91.5	ENSMUSP00000023468	16	96.2

ENSP00000363149	6	ENSTNIP00000002369	9	62.5	ENSTNIP00000021475	11	74.1
ENSP00000347314	13	ENSTNIP00000012814	7	42.3	ENSTNIP00000019362	16	53.6
ENSP00000364054	X	ENSECAP00000002255	X	74.8	ENSECAP00000022481	23	83.5
ENSP00000347721	17	ENSTNIP00000011407	7	46.2	ENSTNIP00000009266	3	50.2
ENSP00000350864	20	ENSECAP00000018782	22	67.6	ENSECAP00000015861	9	72.1
ENSP00000376112	6	ENSTNIP00000015218	14	76.2	ENSTNIP00000022511	10	78.1
ENSP00000346265	19	ENSORLP00000008623	19	55.2	ENSORLP00000020459	23	58.4
ENSP00000344658	10	ENSPTRP00000004729	10	48.3	ENSPTRP00000027970	4	59.8
ENSP00000275200	6	ENSDARP00000068971	20	32.8	ENSDARP00000091247	20	40.3
ENSP00000361998	X	ENSRNOP00000048993	X	48.4	ENSRNOP00000029450	7	63.2
ENSP00000385347	4	ENSTNIP00000016024	20	71.6	ENSTNIP00000006927	18	78.3
ENSP00000382982	13	ENSPTRP00000021340	13	98.9	ENSPTRP00000021312	2b	100
ENSP00000318197	2	ENSPPYP00000014962	2b	97.8	ENSPPYP00000005899	13	99.1
ENSP00000341289	9	ENSCAFP00000028782	9	97.7	ENSCAFP00000027491	20	98.6
ENSP00000264071	19	ENSMODP00000018721	3	93.9	ENSMODP00000021471	1	98.6
ENSP00000361859	X	ENSMMUP00000040914	X	91.3	ENSMMUP00000004663	X	93.4
ENSP00000355332	11	ENSTNIP00000000026	13	80.7	ENSTNIP00000012228	5	90
ENSP00000331500	17	ENSBTAP00000020343	19	65.2	ENSBTAP00000011370	3	70.1
ENSP00000355119	X	ENSMUSP00000073522	X	41	ENSMUSP00000073074	2	50.6
ENSP00000295899	3	ENSBTAP00000043944	22	98.5	ENSBTAP00000012008	17	100
ENSP00000376930	3	ENSTNIP00000021084	9	78.6	ENSTNIP00000017706	11	82
ENSP00000361095	1	ENSTNIP00000009744	15	37.6	ENSTNIP00000005902	1	47.6
ENSP00000329902	7	ENSECAP00000018615	13	15.8	ENSECAP00000019054	11	36.5
ENSP00000234798	16	ENSBTAP00000009636	25	46.7	ENSBTAP00000027425	25	52.7
ENSP00000232496	3	ENSDARP00000096613	22	66.1	ENSDARP00000069296	4	71.6
ENSP00000304908	18	ENSBTAP00000022959	24	52.7	ENSBTAP00000041860	8	62.9
ENSP00000304908	18	ENSCAFP00000027629	7	49.2	ENSCAFP00000004307	11	58.3
ENSP00000304908	18	ENSMUSP00000054909	17	48.9	ENSMUSP00000030051	4	61
ENSP00000307863	19	ENSMMUP00000006397	19	92.8	ENSMMUP00000035632	109	99.5
ENSP00000219638	16	ENSCAFP00000026061	6	83.9	ENSCAFP00000022992	8	90.7
ENSP00000347997	11	ENSCAFP00000033488	21	30.1	ENSCAFP00000002072	1	51.8
ENSP00000366819	13	ENSRNOP00000012842	15	93	ENSRNOP00000008390	3	97.8
ENSP00000367952	10	ENSDARP00000030232	4	43.9	ENSDARP00000026630	25	50.7
ENSP00000251566	4	ENSBTAP00000025005	6	61.1	ENSBTAP00000051040	6	74.2
ENSP00000357040	1	ENSCAFP00000018506	38	51.7	ENSCAFP00000014338	17	73.7
ENSP00000253413	22	ENSTNIP00000011645	13	72.1	ENSTNIP00000014358	19	79.2
ENSP00000361686	10	ENSTNIP00000016146	2	76.2	ENSTNIP00000011469	17	82.7
ENSP00000211998	10	ENSORLP00000011805	15	81.7	ENSORLP00000010700	19	87.6
ENSP00000329565	7	ENSPTRP00000047793	7	92.2	ENSPTRP00000047749	7	96.5
ENSP00000350954	10	ENSORLP00000006281	20	80	ENSORLP00000007775	17	84.8
ENSP00000333595	19	ENSRNOP00000026088	1	44.5	ENSRNOP00000039434	1	48.9
ENSP00000338225	2	ENSORLP00000013194	21	54.1	ENSORLP00000020591	16	57.1
ENSP00000352233	12	ENSPTRP00000007907	12	92.8	ENSPTRP00000057421	11	96
ENSP00000216268	22	ENSMUSP00000035437	15	82.3	ENSMUSP00000111895	12	85.1
ENSP00000232974	3	ENSGALP00000008464	2	77.7	ENSGALP00000039496	27	86.2
ENSP00000366161	9	ENSDARP00000019037	5	66.5	ENSDARP00000022273	10	71.3
ENSP00000311679	9	ENSMUSP00000103178	4	39	ENSMUSP00000086191	17	44.3
ENSP00000311679	9	ENSRNOP00000020207	5	39	ENSRNOP00000039434	1	44.6

Table S5. AmiGO enrichment analysis for 607 detected events, including both artifactual and putative true events. GO biological processes found on AmiGO server with P-value<10-4.

GO Term	GO Term	P-value	N	Sample frequency	Background frequency
GO:0032501	multicellular organismal process	3.58E-43	187	37.9%	12.5%
GO:0048856	anatomical structure development	1.91E-32	138	28.0%	8.6%
GO:0032502	developmental process	2.03E-31	152	30.8%	10.5%
GO:0048731	system development	2.60E-28	121	24.5%	7.4%
GO:0007275	multicellular organismal development	2.89E-28	136	27.6%	9.2%
GO:0065007	biological regulation	3.59E-26	250	50.7%	26.7%
GO:0008150	biological_process	4.54E-26	425	86.2%	63.5%
GO:0050789	regulation of biological process	6.82E-24	237	48.1%	25.4%
GO:0007399	nervous system development	2.98E-23	75	15.2%	3.4%
GO:0050794	regulation of cellular process	3.16E-23	226	45.8%	23.8%
GO:0048869	cellular developmental process	8.34E-23	100	20.3%	6.1%
GO:0009987	cellular process	3.41E-22	360	73.0%	49.9%
GO:0031323	regulation of cellular metabolic process	1.36E-21	136	27.6%	10.8%
GO:0050896	response to stimulus	4.16E-21	203	41.2%	20.8%
GO:0080090	regulation of primary metabolic process	6.41E-21	134	27.2%	10.7%
GO:0009653	anatomical structure morphogenesis	1.24E-20	83	16.8%	4.6%
GO:0071842	cellular component organization at cellular level	2.57E-20	110	22.3%	7.8%
GO:0030154	cell differentiation	3.69E-20	92	18.7%	5.7%
GO:0016043	cellular component organization	1.23E-19	126	25.6%	10.0%
GO:0071841	cellular component organization or biogenesis at cellular level	3.27E-19	112	22.7%	8.3%
GO:0071840	cellular component organization or biogenesis	4.84E-19	129	26.2%	10.5%
GO:0060255	regulation of macromolecule metabolic process	4.89E-19	128	26.0%	10.4%
GO:0051171	regulation of nitrogen compound metabolic process	5.01E-19	114	23.1%	8.6%
GO:0019222	regulation of metabolic process	5.77E-19	139	28.2%	11.9%
GO:0019219	regulation of nucleobase-containing compound metabolic process	7.04E-18	111	22.5%	8.5%
GO:0003008	system process	1.49E-17	63	12.8%	3.1%
GO:0048468	cell development	3.11E-17	63	12.8%	3.1%

GO:0048513	organ development	4.66E-17	85	17.2%	5.5%
GO:0022008	neurogenesis	1.68E-16	53	10.8%	2.3%
GO:0048699	generation of neurons	2.08E-16	51	10.3%	2.1%
GO:0032774	RNA biosynthetic process	5.13E-16	101	20.5%	7.7%
GO:2000112	regulation of cellular macromolecule biosynthetic process	6.67E-16	102	20.7%	7.8%
GO:0006139	nucleobase-containing compound metabolic process	9.51E-16	153	31.0%	15.0%
GO:0051252	regulation of RNA metabolic process	1.90E-15	98	19.9%	7.4%
GO:0016070	RNA metabolic process	1.95E-15	117	23.7%	9.9%
GO:0031326	regulation of cellular biosynthetic process	2.65E-15	105	21.3%	8.4%
GO:0010556	regulation of macromolecule biosynthetic process	2.79E-15	102	20.7%	8.0%
GO:0009889	regulation of biosynthetic process	4.59E-15	105	21.3%	8.4%
GO:0006355	regulation of transcription, DNA-dependent	8.12E-15	95	19.3%	7.2%
GO:0010468	regulation of gene expression	8.23E-15	104	21.1%	8.4%
GO:0050877	neurological system process	9.50E-15	49	9.9%	2.2%
GO:2001141	regulation of RNA biosynthetic process	1.09E-14	95	19.3%	7.3%
GO:0006351	transcription, DNA-dependent	1.78E-14	97	19.7%	7.6%
GO:0030182	neuron differentiation	2.16E-14	46	9.3%	2.0%
GO:0034641	cellular nitrogen compound metabolic process	3.27E-14	160	32.5%	16.7%
GO:0032989	cellular component morphogenesis	1.59E-13	45	9.1%	2.0%
GO:0006807	nitrogen compound metabolic process	2.72E-13	160	32.5%	17.1%
GO:0000902	cell morphogenesis	3.44E-13	43	8.7%	1.8%
GO:0010467	gene expression	3.72E-13	124	25.2%	11.7%
GO:0048666	neuron development	4.24E-13	39	7.9%	1.5%
GO:0030030	cell projection organization	8.30E-13	42	8.5%	1.8%
GO:0006366	transcription from RNA polymerase II promoter	9.41E-13	53	10.8%	2.8%
GO:0044237	cellular metabolic process	5.23E-12	224	45.4%	28.6%
GO:0090304	nucleic acid metabolic process	5.58E-12	127	25.8%	12.5%
GO:0051179	localization	6.58E-12	132	26.8%	13.3%
GO:0023052	signaling	1.04E-11	133	27.0%	13.5%
GO:0048858	cell projection morphogenesis	1.21E-11	33	6.7%	1.2%
GO:0032990	cell part morphogenesis	2.09E-11	33	6.7%	1.2%
GO:0007154	cell communication	3.47E-11	135	27.4%	14.1%
GO:0044260	cellular macromolecule metabolic process	4.06E-11	176	35.7%	20.7%

GO:0034645	cellular macromolecule biosynthetic process	1.00E-10	115	23.3%	11.2%
GO:0009887	organ morphogenesis	3.69E-10	39	7.9%	1.9%
GO:0009059	macromolecule biosynthetic process	3.79E-10	115	23.3%	11.5%
GO:0048523	negative regulation of cellular process	1.68E-09	76	15.4%	6.2%
GO:0000904	cell morphogenesis involved in differentiation	2.58E-09	31	6.3%	1.3%
GO:0051239	regulation of multicellular organismal process	2.59E-09	56	11.4%	3.8%
GO:0007267	cell-cell signaling	3.95E-09	37	7.5%	1.8%
GO:0042060	wound healing	4.10E-09	26	5.3%	0.9%
GO:0009888	tissue development	5.85E-09	47	9.5%	2.9%
GO:0044238	primary metabolic process	5.90E-09	223	45.2%	30.2%
GO:0044249	cellular biosynthetic process	6.53E-09	133	27.0%	14.8%
GO:0048519	negative regulation of biological process	1.56E-08	79	16.0%	6.9%
GO:0007596	blood coagulation	1.57E-08	22	4.5%	0.7%
GO:0031325	positive regulation of cellular metabolic process	1.66E-08	54	11.0%	3.8%
GO:0006810	transport	1.98E-08	109	22.1%	11.3%
GO:0007599	hemostasis	2.00E-08	22	4.5%	0.7%
GO:0048598	embryonic morphogenesis	2.49E-08	29	5.9%	1.2%
GO:0042221	response to chemical stimulus	3.08E-08	68	13.8%	5.6%
GO:0050817	coagulation	3.28E-08	22	4.5%	0.7%
GO:0048667	cell morphogenesis involved in neuron differentiation	3.45E-08	26	5.3%	1.0%
GO:0043170	macromolecule metabolic process	3.70E-08	186	37.7%	24.0%
GO:0051173	positive regulation of nitrogen compound metabolic process	4.62E-08	45	9.1%	2.8%
GO:0048812	neuron projection morphogenesis	4.81E-08	26	5.3%	1.0%
GO:0051234	establishment of localization	5.28E-08	109	22.1%	11.5%
GO:0007389	pattern specification process	5.36E-08	27	5.5%	1.1%
GO:0048522	positive regulation of cellular process	5.86E-08	77	15.6%	6.8%
GO:0006357	regulation of transcription from RNA polymerase II promoter	6.33E-08	42	8.5%	2.5%
GO:0031175	neuron projection development	6.62E-08	29	5.9%	1.3%
GO:0065009	regulation of molecular function	6.90E-08	53	10.8%	3.8%
GO:0009790	embryo development	7.17E-08	40	8.1%	2.3%
GO:0009058	biosynthetic process	7.62E-08	133	27.0%	15.3%
GO:0010604	positive regulation of macromolecule metabolic process	9.41E-08	52	10.5%	3.7%
GO:0048518	positive regulation of biological process	9.47E-08	83	16.8%	7.7%
GO:0009893	positive regulation of metabolic	9.65E-08	54	11.0%	3.9%

	process				
GO:0001501	skeletal system development	1.27E-07	23	4.7%	0.8%
GO:0051254	positive regulation of RNA metabolic process	3.14E-07	41	8.3%	2.6%
GO:0006928	cellular component movement	3.17E-07	41	8.3%	2.6%
GO:0008152	metabolic process	3.17E-07	238	48.3%	34.2%
GO:0010557	positive regulation of macromolecule biosynthetic process	3.22E-07	43	8.7%	2.8%
GO:0007417	central nervous system development	3.23E-07	30	6.1%	1.4%
GO:0045935	positive regulation of nucleobase-containing compound metabolic process	3.49E-07	43	8.7%	2.8%
GO:0045893	positive regulation of transcription, DNA-dependent	3.93E-07	40	8.1%	2.5%
GO:0010628	positive regulation of gene expression	5.28E-07	41	8.3%	2.6%
GO:0051649	establishment of localization in cell	5.98E-07	52	10.5%	3.9%
GO:0031328	positive regulation of cellular biosynthetic process	6.40E-07	44	8.9%	3.0%
GO:0009891	positive regulation of biosynthetic process	8.04E-07	44	8.9%	3.0%
GO:0051716	cellular response to stimulus	8.98E-07	129	26.2%	15.2%
GO:0040011	locomotion	1.14E-06	40	8.1%	2.6%
GO:0050793	regulation of developmental process	1.23E-06	44	8.9%	3.0%
GO:0006996	organelle organization	1.65E-06	62	12.6%	5.3%
GO:0007165	signal transduction	1.93E-06	111	22.5%	12.5%
GO:0065008	regulation of biological quality	2.05E-06	63	12.8%	5.5%
GO:0007600	sensory perception	2.26E-06	22	4.5%	0.9%
GO:0050878	regulation of body fluid levels	2.69E-06	22	4.5%	0.9%
GO:0007166	cell surface receptor linked signaling pathway	3.17E-06	67	13.6%	6.1%
GO:0007409	axonogenesis	3.53E-06	22	4.5%	0.9%
GO:0048646	anatomical structure formation involved in morphogenesis	3.61E-06	32	6.5%	1.8%
GO:0007411	axon guidance	4.81E-06	17	3.4%	0.5%
GO:2000026	regulation of multicellular organismal development	5.48E-06	37	7.5%	2.4%
GO:0003002	regionalization	5.71E-06	21	4.3%	0.8%
GO:0051641	cellular localization	6.89E-06	54	11.0%	4.4%
GO:0007268	synaptic transmission	1.06E-05	23	4.7%	1.0%
GO:0019226	transmission of nerve impulse	1.34E-05	25	5.1%	1.2%

GO:0035637	multicellular organismal signaling	1.34E-05	25	5.1%	1.2%
GO:0007601	visual perception	1.79E-05	14	2.8%	0.4%
GO:0050953	sensory perception of light stimulus	2.04E-05	14	2.8%	0.4%
GO:0023051	regulation of signaling	2.54E-05	54	11.0%	4.6%
GO:0006935	chemotaxis	2.95E-05	22	4.5%	1.0%
GO:0042330	taxis	3.03E-05	22	4.5%	1.0%
GO:0009952	anterior/posterior pattern specification	3.83E-05	16	3.2%	0.5%
GO:0016477	cell migration	4.75E-05	29	5.9%	1.7%
GO:0045595	regulation of cell differentiation	6.46E-05	32	6.5%	2.0%
GO:0006950	response to stress	6.57E-05	69	14.0%	6.8%
GO:0048870	cell motility	7.88E-05	30	6.1%	1.8%
GO:0051674	localization of cell	7.88E-05	30	6.1%	1.8%
GO:0048729	tissue morphogenesis	7.91E-05	23	4.7%	1.1%
GO:0009611	response to wounding	8.31E-05	30	6.1%	1.8%
GO:0051090	regulation of sequence-specific DNA binding transcription factor activity	9.61E-05	17	3.4%	0.6%
GO:0030168	platelet activation	9.94E-05	12	2.4%	0.3%
GO:0044092	negative regulation of molecular function	1.04E-04	24	4.9%	1.2%
GO:0009605	response to external stimulus	1.24E-04	35	7.1%	2.4%
GO:0006195	purine nucleotide catabolic process	1.28E-04	21	4.3%	1.0%
GO:0008219	cell death	1.32E-04	44	8.9%	3.5%
GO:0016265	death	1.39E-04	44	8.9%	3.5%
GO:0048583	regulation of response to stimulus	1.63E-04	58	11.8%	5.4%
GO:0044085	cellular component biogenesis	1.70E-04	49	9.9%	4.2%
GO:0007517	muscle organ development	1.70E-04	18	3.7%	0.7%
GO:0022607	cellular component assembly	1.84E-04	45	9.1%	3.7%
GO:0009719	response to endogenous stimulus	2.11E-04	27	5.5%	1.6%
GO:0071844	cellular component assembly at cellular level	2.30E-04	38	7.7%	2.9%
GO:0072523	purine-containing compound catabolic process	2.53E-04	21	4.3%	1.0%
GO:0033036	macromolecule localization	3.28E-04	49	9.9%	4.3%
GO:0034655	nucleobase-containing compound catabolic process	3.79E-04	22	4.5%	1.1%
GO:0009166	nucleotide catabolic process	3.91E-04	21	4.3%	1.0%
GO:0002009	morphogenesis of an epithelium	4.00E-04	19	3.9%	0.9%
GO:0009203	ribonucleoside triphosphate catabolic process	5.34E-04	19	3.9%	0.9%
GO:0009207	purine ribonucleoside triphosphate catabolic process	5.34E-04	19	3.9%	0.9%
GO:0001763	morphogenesis of a branching structure	5.43E-04	15	3.0%	0.5%
GO:0009146	purine nucleoside triphosphate catabolic process	5.78E-04	19	3.9%	0.9%

GO:0016568	chromatin modification	6.08E-04	19	3.9%	0.9%
GO:0007420	brain development	6.38E-04	21	4.3%	1.1%
GO:0061138	morphogenesis of a branching epithelium	6.46E-04	14	2.8%	0.5%
GO:0009143	nucleoside triphosphate catabolic process	6.57E-04	19	3.9%	0.9%
GO:0009154	purine ribonucleotide catabolic process	6.91E-04	19	3.9%	0.9%
GO:0009261	ribonucleotide catabolic process	7.56E-04	19	3.9%	0.9%
GO:0045944	positive regulation of transcription from RNA polymerase II promoter	8.38E-04	25	5.1%	1.5%
GO:0006915	apoptosis	8.42E-04	40	8.1%	3.3%
GO:0060173	limb development	9.25E-04	12	2.4%	0.3%
GO:0035239	tube morphogenesis	9.38E-04	17	3.4%	0.7%

Table S6. AmiGO enrichment analysis for 250 putative true events. GO biological processes found on AmiGO server with P-value<10-4.

GO Term	GO Term	P-value	N	Sample frequency	Background frequency
GO:0032501	multicellular organismal process	2.02E-13	72	35.1%	12.5%
GO:0050896	response to stimulus	9.04E-12	93	45.4%	20.8%
GO:0008150	biological_process	1.74E-10	178	86.8%	63.5%
GO:0048856	anatomical structure development	2.87E-09	52	25.4%	8.6%
GO:0032502	developmental process	3.95E-09	58	28.3%	10.5%
GO:0048731	system development	3.44E-08	46	22.4%	7.4%
GO:0042060	wound healing	2.23E-07	16	7.8%	0.9%
GO:0007275	multicellular organismal developme	3.85E-07	50	24.4%	9.2%
GO:0050789	regulation of biological process	1.35E-06	93	45.4%	25.4%
GO:0065007	biological regulation	1.68E-06	96	46.8%	26.7%
GO:0071842	cellular component organization at	1.76E-06	44	21.5%	7.8%
GO:0016043	cellular component organization	1.93E-06	51	24.9%	10.0%
GO:0007399	nervous system development	2.03E-06	28	13.7%	3.4%
GO:0048869	cellular developmental process	2.33E-06	38	18.5%	6.1%
GO:0007596	blood coagulation	4.24E-06	13	6.3%	0.7%
GO:0007599	hemostasis	4.92E-06	13	6.3%	0.7%
GO:0030154	cell differentiation	4.92E-06	36	17.6%	5.7%
GO:0009653	anatomical structure morphogenesis	5.54E-06	32	15.6%	4.6%
GO:0050817	coagulation	6.69E-06	13	6.3%	0.7%
GO:0050794	regulation of cellular process	8.93E-06	87	42.4%	23.8%
GO:0071841	cellular component organization or	1.22E-05	44	21.5%	8.3%
GO:0071840	cellular component organization or	1.25E-05	51	24.9%	10.5%
GO:0048468	cell development	2.64E-05	25	12.2%	3.1%
GO:0051179	localization	3.80E-05	58	28.3%	13.3%
GO:0022008	neurogenesis	4.90E-05	21	10.2%	2.3%
GO:0006928	cellular component movement	5.90E-05	22	10.7%	2.6%
GO:0071844	cellular component assembly at cel	9.30E-05	23	11.2%	2.9%
GO:0009987	cellular process	9.41E-05	141	68.8%	49.9%
GO:0048513	organ development	9.48E-05	33	16.1%	5.5%
GO:0050878	regulation of body fluid levels	1.05E-04	13	6.3%	0.9%
GO:0033036	macromolecule localization	2.43E-04	28	13.7%	4.3%
GO:0048699	generation of neurons	3.67E-04	19	9.3%	2.1%
GO:0030182	neuron differentiation	4.44E-04	18	8.8%	2.0%
GO:0023052	signaling	4.45E-04	56	27.3%	13.5%
GO:0022607	cellular component assembly	6.45E-04	25	12.2%	3.7%
GO:0007154	cell communication	6.47E-04	57	27.8%	14.1%
GO:0009611	response to wounding	9.82E-04	17	8.3%	1.8%
