# Supplementary material for: Controversies in modern evolutionary biology: the imperative for error detection and quality control
Source: BMC Genomics. 2012 Jan 4;13:5. doi: 10.1186/1471-2164-13-5 (PMC3311146; doi:10.1186/1471-2164-13-5)
Supplement: Additional file 2 — Examples of erroneous protein sequences and their validation. Example text and figures are provided as a PDF file. [file 1471-2164-13-5-S2.PDF]

Examples of protein sequence errors leading to the detection of artifactual asymmetric evolution after duplication (AED) events

1. Example of a protein sequence error resulting from genome sequencing errors (represented by N characters in the gene sequence).

a) Part of the multiple alignment of homologous proteins from the Ensembl database, showing the predicted deletions in the macaque protein sequence (ENSMMUP00000032519).

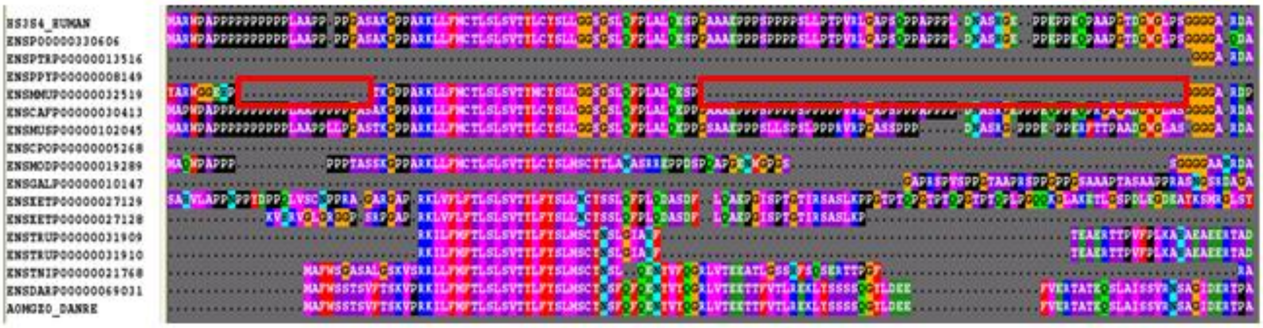

b) The gene sequence corresponding to the ENSMMUP00000032519.

```
>chromosome:MMUL_1:20:24159628:24162238:1
AATTTCAGCGAGGCGATGCACAGACACCCTGCAACCCAGCTTGTCTCTGCTTATTAGGTG
TTCAGAGCGACAATTGTCCCACACTATTTCAGTCCAGGAAACCATGAGCTCCGTTAGTGG
CAATGCCCCCGAAGAGGCGCAGGTGTGTGCACCTGTGATTAAGTGTGTCGAGGATTAAGT
GATTAAGCCTCATCTCTTGGAGCAGAAAGTGTTGTCACCTGGTGATGGGACAGCGGGAAA
AGCTCTGGGGCTGGGAAACCTGGGGGCTTGTGTCAAAGCTCCACCCATCAGGAGCTTCAA
GAGAAGATGGGGGTGGGGGGGGGGTGGCTGGAAAGATGGAAGTTGGGATGGGAAAGCGG
TTGTAGAGAAGGATTCACTCCTGGGCCGGAGGCAGGAGGATATCCCGGGCGAGAGAAGGG
AGGGTCGGGGATGGGCTGAGTTGGAGTCCCAGGGGAAAAGCGGAAGCGAGAGCTTCGTCA
CCCGCTGTCTTCCAGCTCCCGGTGCGCGGCACCGGCGGCTGGCGTTGGGCTCTACCTCTC
TAAAAGTACTGGGGCAAAGGAATGGAGAACACGGCGTCCCGAGCTCCCAAGGGAGGGGAG
TACGCGAGGTGGGGTGGGGAACACCCAAGTGAGTGTATGCTNNNNNNNNNNNNNNNNNNN
NNNNNNNNNNNNNNNNNNNNNNNNNNNNNNNNNNNNNNNNNNNNNNNNNNNNNNNNNNNN
NNNNNNNNNNNNNNNNNNNNNNNNNNNNNNNNNNNNNNNNNNNNNNNNNNNNNNNNNNNN
NNNNNNNNNNNNNNNNNNNNNNNNNNNNNNNNNNNNNNNNNNNNNNNNNNNNNNNNNNNN
NNNNNNNNNNNNTGAGTGTATGCTGGGGGGCTGGGGGGCATGATCTCCGCTCTCCCGGGT
GCCCCAGCCCTAGCGCACGCCTCCGCTCCTGCGCCCCCCTTCGCAGGCGCGCGCGAGGCG
CACTCCCCTTCCCTCGGCGGCGCCGGGCGCGCGCCCGGCCCCCTCCTCCTCCCCTCCGCG
CGTCTCCTCTCTCCCGGCAGAAAGTTAGCAGCGGGGAAGGAACTCGGGGCTGCAACAGCG
CGCGGCGGCGGCGGCAGAGGCTGAAGCAGGAGCCGCAGCGGAGCCGGGGAAGCGGGGGCG
CTGCAGACGGAGCAGGTGCCGCCGGCGGGTCCACGCGCCCCCCTCGGTCCCCTTGCCTGA
GGCTGAGGGGGGGGCACTGGTGCGGGGGCCACCCGGACTCGGCGGGCAGCCTGGGGCGGG
GGGCCATGCGGCCGGGCTCCCNNNNNNNNNNNNNNNNNNNNNNNNNNNNNNNNNNNNNNN
NNNNNNNNNNNNNNNNNNNNNNNNNNNNNNNGCTAAGGGGCCGCCGGCGCGCAAGCTGCT
TTTTATGTGCACCTTGTCCCTGTCTGTCACCTACATGTGCTACAGCCTCCTGGGCGGCTC
GGGCTCCCTGCAATTCCCCCTGGCGCTGCAGGAGTCGCCGGGCACCGCCGCCGAGCCCCC
GCCGAGCCCGCCGCCACCCTCTCTGCTGCCTCCCCCCGTGCGCCTCGGCGCCCCCTCGCA
GCCACCCGCGCCGCCGCCGCTGGACAACGCGAGCCGCGGGGAGCCGCCCGAGCCCCCTGA
GCAGCCCGCCGCCCCCGGGACCGACGGCTGGGGGCTGCCGAGCGGCGGCGGAGGCGCCCG
GGACCCCTGGCTCCGGACCCCGCTGGCCCCCAGCGAGATGATCACGGCTCAGAGCGCGCT
GCCAGAGAGGGAAGCGCAGGAGTCCAGCACCACCGACGAGGATCTCGCAGGCCGGAGAGC
GGCCAACGGGAGCAGCGAGAGGGGCGGCGCCGTCAGCACCCCTGACTATGGGGAGAAGAA
GCTGCCACAGGCGCTCATCATCGGGGTCAAGAAAGGAGGGACCCGCGCGCTGCTGGAGGC
GATCCGCGTACACCCGGACGTGCGGGCGGTGGGCGTAGAGCCGCACTTCTTCGACAGGAA
CTACGAGAAGGGGTTGGAGTGGTACAGGTAGGACTCTGGGCTCCGCGGGCTGGTGGAGAC
GCGTGGGGGAGACGCGGAGGGGAAGCCGCGGCTTTCCACGCCCTTCGAGCATCCAGGCAC
CGTCCCGAGAGGCCCAAGCCCCCGCGAGGGCTCTGCAAACCCTGGCGGCTTTGCTCAGGG
GGATAGGCTGAGAGGGCTGGACTCCAGCGAAAGATCACTTTATTTCAGGGCGAGGAGAGG
AGGTGTCACCCTGCCCTGCCTCCCGCGCTCCTCATCCAAGGAGGTGCTGTCTGAATCTGC
CCAGCTCCCAGCCTGGGAATCCCCAGCCCTCGTGCCTGCTGGGTGTTTCCGAACCCAGGC
TCTTGCGGGATTCTGGGATTCTGGGCTGAGGACGCTGAGGAGTGAGACAGGATGGCTAAA
TTGACTAAGGGGATTTGAGGTCCCCTGCAATCTCTTAAAATCACCCTCAAACGCATTTGC
GTGGCTGGAATTCAACTTGAGTGTGTTAAGGTCAGAGCAAAATGAATAGGGAACAGTTAC
AAAGATCATGCTGGCGTTTTGGCTTTCTAGTGAAGAAAGGATGCCTCCCACCTCCATAAC
TTTCCATCCCCCTGGACTGAATGAGCACGGA
```

2. Example of an N-terminal extension that decreases the genetic distance between the human reference sequence (ENSP00000376478) and the syntenic sequence in Tetraodon (ENSTNIP00000019604).

a) Part of the multiple alignment of homologous proteins from the Ensembl database, showing the sub-group of sequences from fish and the predicted N-terminal extension.

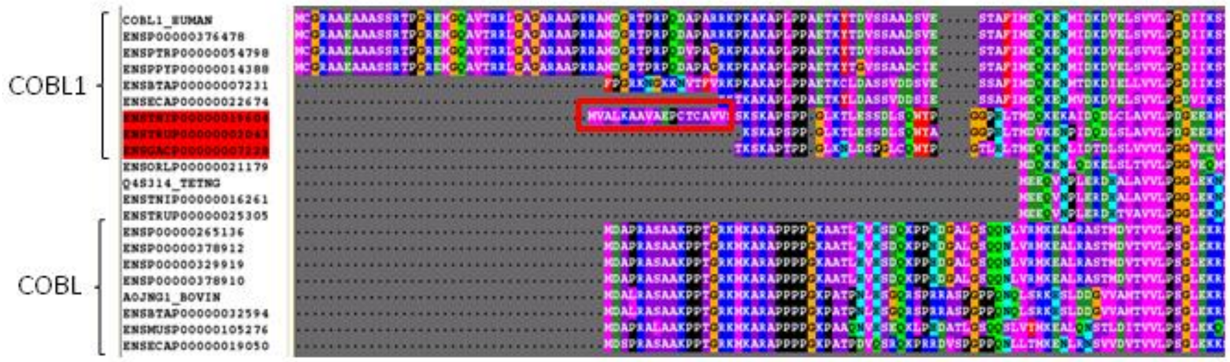

b) The transcript evidence in Ensembl for this protein is A0JNG1.1: the cordon-bleu homolog (COBL) from *Bos taurus*, which is a paralog of ENSTNIP00000019604.

```
ENSTNIP00000019604  1      MVALKAAVAEPCTCAVVSSKSKAPSPPG---LKTLESSDLS--QWYPGGPHLTMDQKEKA
                           M AL+A+ A+P T   +  K++AP PPG      L S   S  +  PG P   + +K
A0JNG1.1            1      MDALRASAAKPPTGRKM--KARAPPPPGKPATPNLHSGQRSPRRASPGPPQNQLSRKHSL


ENSTNIP00000019604  56     IDQDLCLAVVLPDGEERMTTVHGSKPLMDLLVTLCVQYHLNPSSYTLELVTANRKNT-KL
                            D  + + VVLP G E+ + V+GS  +MDLLV LC+Q HLNPS++ LE+ ++  +
A0JNG1.1            59     DDGVVAMTVVLPSGLEKRSVVNGSHAMMDLLVELCLQNHLNPSNHALEIRSSETQQPLNF
```

3. An example of alternative splicing variants predicted for different organisms, resulting in a C-terminal extension error prediction in zebrafish (ENSDARP00000069381).

a) Two different mRNAs are available for zebrafish: Q9PVD8_DANRE and Q90Z68_DANRE. Q4RA24_TETNG is a fragment derived from a whole genome shotgun sequence. In the Ensembl database, only the longer transcript is predicted for zebrafish (supported by Q90Z68_DANRE), while only the shorter transcript is predicted for tetraodon (supported by Q9PVD8_DANRE).

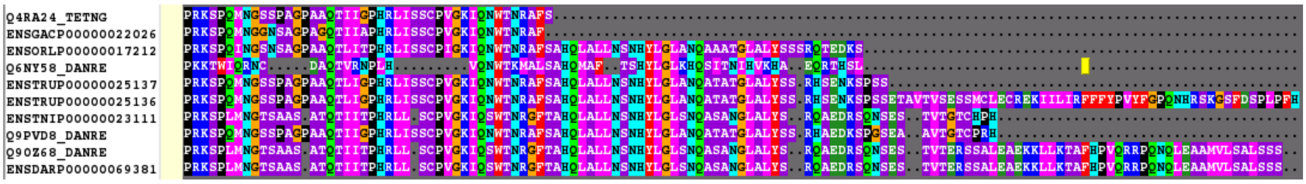

b) The C-terminal extension corresponds to two exons in the zebrafish sequence.

```
>ENSDARP00000069381

MSFPQLGYQYIRPIYSQDRQGIGSARAGTDLSPSGALSNVLSTMYGSPFAAAQSYGAFLP
YSNDLSIFNQLGAQYELKDSPGVQHPGFAHHHPAFYPYGQYQFGDPSRPKNATRESTSTL
KAWLSEHRKNPYPTKGEKIMLAIITKMTLTQVSTWFANARRRLKKENKMTWTPRSRTDEE
GNVYNSDHEGDDGDKREDEEEIDLENIDTENIENKDDLDEQDELHSDLKLDGRSDSEISD
GYEDLQGPEQRLFKAMVKDGKEIHGDRAEHFHHHSHHHHLHHNSLEQANGEPVKINQAII
NSPPSENNPPPAPKPKIWSLAETATTPDNPRKSPLMNGTSAASATQTIITPHRLLSCPVG
KIQSWTNRGFTAHQLALLNSNHYLGLSNQASANGLALYSRQAEDRSQNSESTVTERSSAL
EAEKKLLKTAFHPVQRRPQNQLEAAMVLSALSSS
```
